# The Quantum Transport of Dirac Fermions in Selected Graphene Nanosystems Away from the Charge Neutrality Point

**DOI:** 10.3390/ma18092036

**Published:** 2025-04-29

**Authors:** Adam Rycerz

**Affiliations:** Institute for Theoretical Physics, Jagiellonian University, Łojasiewicza 11, PL-30348 Kraków, Poland; rycerz@th.if.uj.edu.pl

**Keywords:** graphene, shot noise, tight-binding model, Landauer–Büttiker formalism

## Abstract

The peculiar electronic properties of graphene, including the universal *dc* conductivity and the pseudodiffusive shot noise, are usually found in a small vicinity close to the charge neutrality point, away from which the electron’s effective mass raises, and nanostructures in graphene start to behave similarly to familiar Sharvin contacts in semiconducting heterostructures. Recently, it was pointed out that as long as abrupt potential steps separate the sample area from the leads, some graphene-specific features can be identified relatively far from the charge neutrality point. These features include greater conductance reduction and shot noise enhancement compared to the standard Sharvin values. The purpose of this paper is twofold: First, we extend the previous analysis based on the effective Dirac equation, and derive the formulas that allow the calculation of the arbitrary charge transfer cumulant for doped graphene. Second, the results of the analytic considerations are compared with numerical simulations of quantum transport on the honeycomb lattice for selected nanosystems for which considerations starting from the Dirac equation cannot be directly adapted. For a wedge-shaped constriction with zigzag edges, the transport characteristics can be tuned from graphene-specific (*sub-Sharvin*) values to standard Sharvin values by varying the electrostatic potential profile in the narrowest section. A similar scenario is followed by the half-Corbino disk. In contrast, a circular quantum dot with two narrow openings showing a mixed behavior appears: the conductance is close to the Sharvin value, while the Fano factor approaches the value characterizing the symmetric chaotic cavity. Carving a hole in the quantum dot to eliminate direct trajectories between the openings reduces the conductance to sub-Sharvin value, but the Fano factor is unaffected. Our results suggest that experimental attempts to verify the predictions for the sub-Sharvin transport regime should focus on systems with relatively wide openings, where the scattering at the sample edges is insignificant next to the scattering at the sample–lead interfaces.

## 1. Introduction

There are few phenomena in nature for which the results of measurements of physical quantities are given directly by the fundamental constants of nature, leaving even the question of the actual number of fundamental constants open [1,2]. In the second half of the last century, two phenomena from this group were discovered and theoretically described: the quantum Hall effect [3,4,5,6] and the Josephson effect [7], which are currently used as the basis for the standards of units of resistance and electric voltage in the SI system, i.e., the ohm [8] and the volt [9]. The discovery of the two-dimensional allotrope of carbon, graphene, made at the beginning of the 21st century [10,11], allowed for the improvement of the ohm standard based on the quantum Hall effect [8]. (Some peculiar features of the Josephson effect in graphene were also pointed out [12,13].) Moreover, it turned out that several material characteristics of graphene, such as the conductivity [14,15] or visible light absorption [16,17], are given by the fundamental constants or dimensionless numerical coefficients. The sub-Poissonian shot noise (quantified by the Fano factor F=1/3) [18,19,20,21] and the anomalous Lorentz number [22,23,24,25] for charge-neutral graphene can also be regarded as examples of such characteristics. Although measurements of these quantities with metrological accuracy are not possible yet, the scientific community have undoubtedly gained unique opportunities to test a theoretical model, which is the effective two-dimensional Dirac–Weyl equation for monolayer graphene [26,27]. Thus, in graphene, the theoretical perspective complements the device-oriented research avenue [28,29,30,31,32].

The author and Witkowski have recently found, using the effective Dirac equation, that for doped graphene samples of highly-symmetric shapes (namely, the rectangle with smooth edges and the Corbino disk) the conductance is reduced, whereas the shot noise is amplified when compared to standard Sharvin values [33,34]. The reduction (or amplification) is maximal when abrupt potential steps separate the sample area from the leads; for instance, the conductance $G\approx \left(\pi /4\right)\;G_{\mathrm{Sharvin}}$ (with $G_{\mathrm{Sharvin}}=g_{0}k_{F}W/\pi$ [35,36,37,38], $g_{0}$ the conductance quantum, $k_{F}$ the Fermi wavenumber, and *W* the sample width (For the disk geometry, the width *W* needs to be replaced with the inner lead circumfence $2\pi R_{i}$)) for the rectangle or the narrow disk (i.e., the inner-to-outer radii ratio $R_{i}/R_{o}\approx 1$), $G\approx \left(4-\pi \right)\;G_{\mathrm{Sharvin}}$ for the wide-disk limit ($R_{i}/R_{o}\ll 1$); the Fano factor F≈1/8 for the rectangle or the disk with $R_{i}/R_{o}\approx 1$, F≈0.1065 for the disk with $R_{i}/R_{o}\ll 1$. When the potential profile gets smoothed, the above-listed *sub-Sharvin* values evolve towards $G\approx G_{\mathrm{Sharvin}}$ and the Fano factor approaches the ballistic value of F≈0. Later, the discussion in analytical terms was extended on the nonzero magnetic field case [39,40], showing that in a doped disk with $R_{i}/R_{o}\approx 1$, the vanishing conductance G→0 (notice that in the disk geometry, the edge states are absent and the current is blocked at a sufficiently high field except from narrow resonances via Landau levels [41,42,43,44,45]) is accompanied by a non-trivial value of F≈0.55, evolving towards the Poissonian limit of F→1 for $R_{i}/R_{o}\ll 1$.

**Figure 1:**
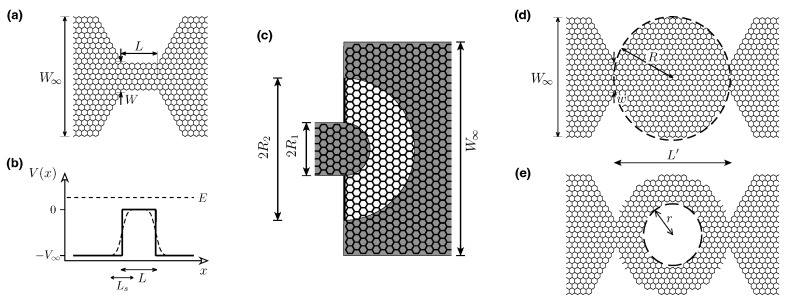
(**a**–**e**) Systems studied numerically in the work (schematic). (**a**) Constriction with zigzag edges containing a narrow rectangular section of the width *W* and the length *L*. (**b**) Corresponding potential profile. (**c**) Half Corbino disk (white area) with the inner radii $R_{1}$ and the outer radii $R_{2}$ attached to doped graphene leads with armchair edges (shaded areas). (**d**) Circular quantum dot of the radii *R*. The electrostatic potential profile is the same as in (**b**), but the steps are placed in the two narrowest sections of *w* width at a distance $L^{\prime }$. (**e**) Circular quantum dot with a circular hole of the radii *r* in the center and the remaining parameters same as in (**d**).

The purpose of this paper is to continue the discussion on the sub-Sharvin transport regime in graphene by going beyond the effective Dirac equation. In particular, we address the question of how realistic (irregular) edges of a nanosystem that are carved out of the honeycomb lattice affect transport characteritics. For this purpose, we perform computer simulations of quantum transport for selected systems containing up to 336,000 lattice sites, which are depicted schematically in Figure 1 and modeled within the tight-binding Hamiltonian. The results show that sub-Sharvin characteristics are closely reconstructed for relatively short and wide systems; for longer and more complex systems with multiple constrictions, some less obvious scenarios (including the sub-Sharvin conductance accompanied by the shot noise power resembling a chaotic cavity) can be observed.

The remaining parts of the paper are organized as follows. In Section 2, we present the Landauer–Büttiker formalism for a generic nanoscopic system and the key literature results following from the effective Dirac equation for graphene at the charge neutrality point as well as in the sub-Sharvin regime. The analytical technique, allowing the calculation of higher charge transfer cumulants for graphene at and away from the charge neutrality point, is also presented in Section 2. Statistical distributions of transmission probabilities for different quantum transport regimes, including the sub-Sharvin transport regime in graphene, are described in Section 3. The tight-binding model of graphene and our main numerical results concerning the conductance and the Fano factor for selected nanosystems (see Figure 1) are presented in Section 4; the details of the computer simulation of quantum transport are given in Appendix A. The conclusions are given in Section 5.

## 2. Landauer–Büttiker Transport in Nanoscopic Systems and Graphene

### 2.1. Remark on the Origin of Zero-Temperature Landauer–Sharvin Resistance

First, let us look for a concise answer to the following question: *Where does electrical resistance come from at absolute zero?*

In the familiar Drude model of electrical conduction [46], electrons are assumed to constantly bounce between heavier, stationary lattice ions, allowing one to express the material specific resistivity as a function of the electron’s effective mass, velocity, and the mean free path. In the quantum-mechanical decription of solids, the Drude model provides a reasonable approximation as long as the Fermi wavelength remains much shorter than the electron’s mean free path and the conductor size.

**Figure 2:**
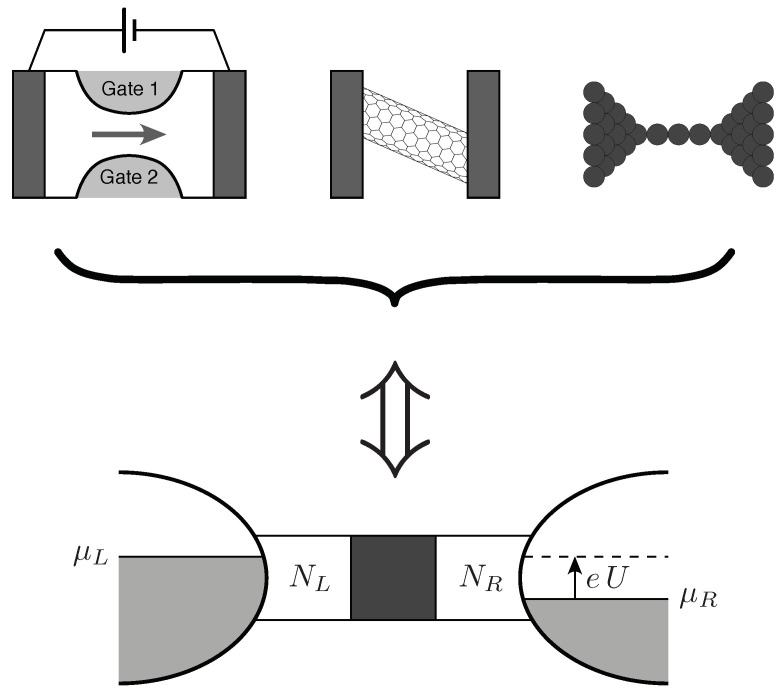
Physical suppositions behind the Landauer–Büttiker formalism. Top: Basic nanoscopic systems; from left to right: a quantum point contact (QPC) in semiconducting heterostructure, a carbon nanotube, and a monoatomic quantum wire (each system is contacted by two electrodes and connected to a voltage source driving a current, as shown for QPC). Bottom: A theoretical model, containing the two macroscopic reservoirs (left and right) with fixed chemical potentials ($\mu_{L}$, $\mu_{R}$), waveguides with their numbers of normal modes ($N_{L}$, $N_{R}$), and the central region (dark square) for which transmission probabilities ($T_{n}$) need to be determined by solving a relevant quantum-mechanical wave equation.

The picture above changes substantially when electic charge flows through a nanoscopic system, such as in quantum point contact in a semiconducting heterostructure [36], a carbon nanotube [47], or a monoatomic quantum wire [48] (see Figure 2, top part). Assuming for simplicity that such a system has no internal degrees of freedom leading to the degeneracy of quantum states, in other words—that in a sufficiently small energy range ΔE, we have at most one quantum state (level)—we note that the time of flight of an electron through the system is limited from below by the time–energy uncertainty relation

(1) $$\Delta t⩾\frac{\hbar }{\Delta E}.$$

Next, by linking the energy range ΔE with the electrochemical potential difference in macroscopic electrodes (reservoirs) connected to the nanoscopic system (see Figure 2, bottom part), we can write

(2) $$\Delta E=\mu_{L}-\mu_{R}=eU,$$

where *U* denotes the difference in electrostatic potential on both sides of the system, and is the elementary charge (without sign). Combining the above equations, we obtain the limit for the electric current flowing through the system

(3) $$I=\frac{e}{\Delta t}⩽\frac{e^{2}}{\hbar }U,$$

which means that the electrical conductivity

(4) $$G=\frac{I}{U}⩽\frac{e^{2}}{\hbar }.$$

We thus see that the uncertainty principle of energy and time leads to a finite value of the conductivity, and therefore to a nonzero value of the electrical resistance in a nanoscopic system. By rigorous derivation, the upper bound in Equation (4) is replaced by $e^{2}/h$, introducing the Landauer–Sharvin resistance in noninteracting electron systems [35,49,50]. Obviously, many-body effects may alter this conclussion substantially [51]. For instance, the resistivity of a graphene sample may drop below the Landauer–Sharvin bound due to hydrodynamic effects [52]. In twisted bilayer graphene, both the interaction-driven insulating and superconducting (i.e., resistance-free) phases were observed [53,54,55]. These issues are, however, beyond the scope of the present work.

### 2.2. The Landauer–Büttiker Formula

At a temperature close to absolute zero (T→0) and in the limit of linear response, i.e., the situation in which the electrochemical potential difference also tends to zero ($\mu_{L}-\mu_{R}=eU\to 0$), it can be shown that the electrical conductivity of a nanoscopic system is proportional to the sum of transition probabilities for the so-called normal modes in the leads [56],

(5) $$G=g_{0}\sum_{n}T_{n}\left(E_{F}\right),$$

where $g_{0}$ denotes the conductance quantum; namely, $g_{0}=2e^{2}/h$ for systems exhibiting spin degeneracy (for graphene, we have $g_{0}=4e^{2}/h$ due to the additional degeneracy—called valley degeneracy—related to the presence of two nonequivalent Dirac points in the dispersion relation). The probabilities ($T_{n}$) are calculated by solving (exactly or approximately) the corresponding wave equation (Schrödinger or Dirac) for a fixed energy, which, given the assumptions made, can be identified with the Fermi energy $E_{F}$. Importantly, we perform the calculations under the additional assumption that there are so-called waveguides between the macroscopic reservoirs and the nanoscopic system, for which we can provide (for a fixed value of $E_{F}$) solutions in the form of propagating waves, the number of which is $N_{L}$ or $N_{R}$ for the left or right waveguide, respectively (see Figure 2). We also assume that any wave incident on a waveguide–reservoir interface, coming from the system, is always absorbed in the reservoir (for the discussion of possible alternative assumptions, see Ref. [50]).

It is worth noting that the sum appearing in Formula (5) is the trace of the transmission matrix, the value of which does not depend on the choice of the basis; therefore, it can be expected that the result does not depend on how precisely we construct the aforementioned waveguides, which, it is worth emphasising, are an auxiliary construction that usually has no direct physical interpretation (for the same reason, the result will be the same whether we consider scattering from left to right or in the opposite direction.) In the context of graphene, it was shown that for various types of waveguides, including normal conductors modeled by square lattices, a broad window of parameters can be identified such that the waveguides appear transparent, i.e., the transport properties are governed by the central region [18,57,58].

As mentioned above, the details of the calculations (or computer simulations) leading to the determination of the probability values ($T_{n}$) will depend on the geometry of the system under consideration. If waveguides are modeled as strips of fixed width (*W*), at the edges of which the wave function disappears, the normal modes have the form of plane waves (Strictly speaking, these solutions take the form of linear combinations of two plane waves (with positive and negative $k_{y}$), producing standing waves in the *y* direction, but this detail is irrelevant to the calculations presented below), for which the longitudinal component of the wave vector ($k_{x}$) is continuous and the normal component ($k_{y}$) is quantized according to the rule

(6) $$k_{y}^{\left(n\right)}=\frac{\pi n}{W},\;\;\;\;n=1,2,\ldots .$$

The calculations are particularly simple in cases where the central region (marked with a dark square in Figure 2) differs from the leads only in that it contains an electrostatic potential that depends on the *x* coordinate (oriented along the main axis of the system), for example, in the form of a rectangular barrier. Then, the transmission matrix has a diagonal form (no scattering between normal modes occurs), and in special cases, such as the rectangular barrier mentioned above, but also, e.g., the parabolic potential considered by Kemble in 1935 [59], it is possible to provide compact analytical formulas.

We will not present the exact results here, but only point out that for solutions obtained by the mode-matching method for the Schrödinger equation, one can write approximately

(7) $$T_{n}=T\left(k_{y}^{\left(n\right)}\right)\approx \left\{\begin{array}{c} 1\;\;\mathrm{if}\;\;k_{y}\leq k_{F},\\ 0\;\;\mathrm{if}\;\;k_{y}>k_{F}, \end{array}\right.$$

which we write more briefly as $T_{n}\approx \Theta \left(k_{F}-k_{y}^{\left(n\right)}\right)$, with Θ(x) denoting the Heaviside step function. In Equation (7), we introduce the wave vector $k_{F}$ corresponding to the Fermi energy $E_{F}$ (assuming for simplicity that the dispersion relation is isotropic) calculated with respect to the top of the potential barrier in the central region. Furthermore, assuming that there are many modes for which $k_{y}^{\left(n\right)}<k_{F}$ (which occurs if $k_{F}W\gg 1$), and therefore the summation in Equation (5) can be replaced to a good approximation by integration, we obtain—via Equations (6) and (7)—the result known in the literature as the Sharvin conductance [35]

(8) $$G_{\mathrm{Sharvin}}\approx g_{0}\frac{k_{F}W}{\pi }.$$

Importantly, the reasoning leading to Equation (8) can be relatively easily applied to the case where the electrostatic potential in the central region is approximately constant and the width of the conducting region is a function of the position along the longitudinal axis (*x*), changing slowly enough that the scattering between normal modes can be neglected. The above-mentioned case is the so-called quantum point contact (QPC), shown schematically in Figure 2 (top part), which can be realized in semiconductor heterostructures hosting a two-dimensional electron gas (2DEG) [56].

In contrast to bulk systems, for which the semiclassical Drude–Boltzmann approach works relatively well [60,61], nanosystems with spatially confined electrons exhibit several quantum effects that can be much better grasped within the Landauer–Büttiker formalism. Additionally, transport in graphene at low carrier concentration is governed by evenescent modes, the inclusion of which in the Drude–Boltzmann description is rather problematic [24]. For instance, Yoshino and Murata [22] assumed linear relaxation time on energy dependence, leading to nonzero conductivity at the charge neutrality point, but the physical reasoning behind such an assumption seems unclear. On the other hand, the Landauer–Büttiker formalism naturally includes both evanescent and propagating solutions, allowing to describe the phenomena such as the universal minimal conductivity of monolayer graphene (further details are given in Section 2.5).

### 2.3. Shot Noise and Counting Statistics

The second quantity besides electrical conductivity that characterizes nanoscopic systems at temperatures close to absolute zero is the shot noise power. For the sake of brevity, let us point out the basic facts: First, the electric charge *Q* flowing through the system shown schematically in Figure 2 (lower part) in a short time interval Δt is a random variable. Second, the expectation value of such a variable is closely related to the electrical conductivity *G* in the linear response limit,

(9) 〈Q〉=GUΔt(U→0).

The reason the measured value of *Q* fluctuates at successive time intervals is due to the discrete (granular) nature of the electric charge.

Assuming (for the moment) that electrons jump from one reservoir to another completely independently, we conclude that the charge flow is a Poisson process, or more precisely, that the quantity Q/e follows the Poisson distribution; the variance is therefore proportional to the expectation value given by Equation (9),

(10) $${\langle Q^{2}-{\langle Q\rangle }^{2}\rangle }_{\mathrm{Poisson}}=e\langle Q\rangle =eU\Delta tg_{0}\sum_{n}T_{n}.$$

More generally, the *m*-th central moment can be written as

(11) $${\langle \langle Q^{m}\rangle \rangle }_{\mathrm{Poisson}}\equiv {\langle {\left(Q-\langle Q\rangle \right)}^{m}\rangle }_{\mathrm{Poisson}}=e^{m-1}\langle Q\rangle ,$$

with the integer m⩾1.

The Fano factor, quantifying the shot noise power, is defined as the ratio of the actual measured variance of the charge flowing through the system to the variance given by Equation (10), or more precisely

(12) $$F=\frac{\langle Q^{2}-{\langle Q\rangle }^{2}\rangle }{{\langle Q^{2}-{\langle Q\rangle }^{2}\rangle }_{\mathrm{Poisson}}}=1-\frac{\sum_{n}T_{n}^{2}}{\sum_{n}T_{n}}.$$

(For compact derivation, see e.g., Ref. [56]). In the following, we have limited our considerations to long time intervals such that eUΔt≫ℏ; hence, *F* characterizes the zero-frequency noise, not to be confused with the celebrated 1/f noise in electronic systems [62]. A generalization of Equation (12) for finite times (and nonzero temperatures) is also possible [63].

In particular, it follows from Equation (12) that the Poisson limit (F→1) is realized in the case of a tunnel junction, for which we have $T_{n}\ll 1$ for each *n*. This is a completely different case than the ballistic system considered above, which exhibits Sharvin conductance; then, replacing the summation with integration as before and using the approximation given by Equation (7), we obtain

(13) $$F_{\mathrm{Sharvin}}\approx 1-\frac{\int dk_{y}{\left(\Theta (k_{F}-k_{y})\right)}^{2}}{\int dk_{y}\;\Theta \left(k_{F}-k_{y}\right)}\approx 0.$$

In general, for fermionic systems, we always have 0<F<1; the factor $1-T_{n}$ appearing in the numerator in Equation (12) is a consequence of the Pauli exclusion principle. In the case of the idealized ballistic system, we have F=0—see Equation (13)—which means that the electron count (Q/e) does not fluctuate with time. One could say that the electrons avoid each other so much that they “march” at equal intervals. (Of course, this is only possible at absolute zero temperature, otherwise additional thermal noise appears, i.e., the Nyquist–Johnson noise proportional to the conductivity value, whose influence we have ignored here; see Ref. [56]).

In an attempt to determine higher charge cumulants, it is convenient to introduce characteristic function

(14) $$\Lambda \left(\chi \right)=\langle \;\exp(i\chi Q/e)\;\rangle ,$$

such that

(15) $$\langle \langle Q^{m}\rangle \rangle \equiv \langle {\left(Q-\langle Q\rangle \right)}^{m}\rangle =e^{m}{\left.\frac{\partial^{m}\ln\Lambda \left(\chi \right)}{\partial {\left(i\chi \right)}^{m}}\right|}_{\chi =0}.$$

Assuming U>0 for simplicity, we arrive at the Levitov–Lesovik formula [56,63]

(16) $$\ln\Lambda \left(\chi \right)=\frac{g_{0}U\Delta t}{e}\sum_{n}\ln\left[1+T_{n}\left(e^{i\chi }-1\right)\right],$$

expressing the full counting for noninteracting fermions.

The substitution of the above into Equation (15) with m=1 and m=2 reproduces, respectively, Equations (5) and (12). Analogously, for m=3 and m=4, we obtain

(17) $$\begin{array}{cc} R_{3} & \equiv \frac{\langle \langle Q^{3}\rangle \rangle }{{\langle \langle Q^{3}\rangle \rangle }_{\mathrm{Poisson}}}=\left(\sum_{n}T_{n}-3\;\sum_{n}T_{n}^{2}+2\;\sum_{n}T_{n}^{3}\right)/\sum_{n}T_{n}, \end{array}$$

(18) $$\begin{array}{cc} R_{4} & \equiv \frac{\langle \langle Q^{4}\rangle \rangle }{{\langle \langle Q^{4}\rangle \rangle }_{\mathrm{Poisson}}}=\left(\sum_{n}T_{n}-7\;\sum_{n}T_{n}^{2}+12\;\sum_{n}T_{n}^{3}-6\;\sum_{n}T_{n}^{4}\right)/\sum_{n}T_{n}. \end{array}$$

For the Sharvin regime, see Equation (7),

(19) $${\left(R_{3}\right)}_{\mathrm{Sharvin}}\approx {\left(R_{4}\right)}_{\mathrm{Sharvin}}\approx 0.$$

### 2.4. Scattering of Dirac Fermions in Two Dimensions

Using the introductory information gathered above, we will now calculate—with some additional simplifying assumptions—the electrical conductivity as well as the higher charge cimulants of a graphene strip. The effective wave equation for itinerant electrons in this two-dimensional crystal is the Dirac–Weyl equation, the detailed derivation of which can be found, e.g., in Katsnelson’s textbook [15], and which can be written in the form

(20) $$\left[v_{F}\;p\cdot \sigma +V\left(x\right)\right]\Psi =E\Psi ,$$

where the energy-independent Fermi velocity is given by $v_{F}=\sqrt{3}t_{0}a/\left(2\hbar \right)$, where $t_{0}\approx 2.7\;\mathrm{eV}$ denotes the nearest-neighbor hopping integral in the graphene plane and a=0.246nm is the lattice constant (as a result, the approximate value of $v_{F}$ is about $10^{6}\;m/s$, which is several times lower than typical Fermi velocities in metals). The remaining symbols in Equation (20) are the quantum mechanical momentum operator $p=-i\hbar \left(\partial_{x},\partial_{y}\right)$ (the notation $\partial_{j}$ here means differentiation with respect to the selected coordinate, j=x,y), $\sigma =\left(\sigma_{x},\sigma_{y}\right)$ is a vector composed of Pauli matrices (It should be noted that Equation (20) is used for the *K*-valley neighborhood in the dispersion relation; for the K′-valley the matrix $\sigma_{y}$ should be replaced by $\sigma_{y}^{★}=-\sigma_{y}$), and the electrostatic potential energy V(x) is assumed to depend only on the position along the principal axis of the system.

The above assumptions imply that we can look for solutions to Equation (20) in the form of a two-component (i.e., spinor) wave function

(21) $$\Psi =\left(\begin{array}{c} \phi_{a}\\ \phi_{b} \end{array}\right)e^{ik_{y}y},$$

where $\phi_{a}$ and $\phi_{b}$ are functions of *x*. By substituting the above ansatz into Equation (20), we obtain a system of ordinary differential equations

(22) $$\begin{array}{cc} \phi_{a}^{\prime } & =k_{y}\phi_{a}+i\frac{E-V(x)}{\hbar v_{F}}\phi_{b}, \end{array}$$

(23) $$\begin{array}{cc} \phi_{b}^{\prime } & =i\frac{E-V(x)}{\hbar v_{F}}\phi_{a}-k_{y}\phi_{b}, \end{array}$$

where the primes on the left-hand side denote derivatives with respect to *x*. We see that in the system of Equations (22) and (23), the quantities $k_{y}$ and *E* play the role of parameters on which the solutions depend (in the following, when calculating, among others, the electrical conductivity, we will identify the electron’s energy with the Fermi energy by setting $E=E_{F}$).

**Figure 3:**
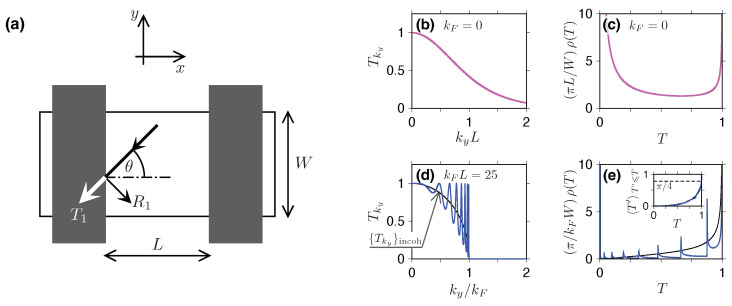
(**a**) Rectangular graphene sample (white area) of the width *W* contacted to the leads (dark areas) at a distance *L*. The coordinate system (x,y) is also shown. Scattering of Dirac electrons at a sample–lead interface for the incident angle θ is characterized by the transmission ($T_{1}$) and the reflection ($R_{1}$) coefficients given by Equation (29). (**b**) Transmission probability for a double barrier [see Equation (30)] as a function of the transverse momentum $k_{y}$ and (**c**) the corresponding distribution of transmission probabilities at the Dirac point $k_{F}=0$ (with $k_{F}=\left|E\right|/\hbar v_{F}$). (**d**,**e**) Same as (**b**,**c**) but the doping is fixed at $k_{F}L=25$. Blue lines represent the exact results, black lines depict the approximation ${\left\{T_{k_{y}}\right\}}_{\mathrm{incoh}}$ given by Equation (41). Inset in (**e**) shows the integrated distribution ${\langle T^{\prime }\rangle }_{T^{\prime }⩽T}=\left(\pi /k_{F}W\right)\int_{0}^{T}dT^{\prime }T^{\prime }\rho \left(T^{\prime }\right)$ for both the exact ρ(T) [blue line] and the approximation given by Equation (61) [black line]. The sub-Sharvin value of 〈T〉=π/4 is depicted with a dashed horizontal line.

At this point, it is worth commenting on the problem of quantizing the value of the transverse momentum ($k_{y}$) in Equations (22) and (23). Assuming that the component of the current density perpendicular to the axis of the graphene strip disappears at its edges (i.e., for y=0 and y=W, see Figure 3a), which is known as the so-called mass confinement [64], we obtain a slightly different quantization than in the case of the Schrödinger system; see Equation (6), namely

(24) $$k_{y}^{\left(n\right)}=\frac{\pi (n+1/2)}{W},\;\;\;\;n=0,1,2,\ldots .$$

In practice, however, the assumptions made in the following part mean that when calculating measurable quantities (*G*, *F*, etc.) we will approximate the sums appearing in Equations (5), (12), (17), and (18), with integrals with respect to $dk_{y}$; the quantization change described above is therefore insignificant for further considerations.

The solution of the system of Equations (22) and (23) is particularly simple in this case if the electrostatic potential energy, i.e., the function V(x), is piecewise constant. Then, the solutions in individual sections (i.e., areas where V(x) is constant) have the form of plane waves. For instance, for E>V(x) waves traveling in the positive (+) and negative (−) directions along the *x* axis, these are as follows

(25) $$\phi^{\left(+\right)}=\left(\begin{array}{c} 1\\ e^{i\theta } \end{array}\right)e^{ik_{x}x},\;\;\;\;\phi^{\left(-\right)}=\left(\begin{array}{c} 1\\ -e^{-i\theta } \end{array}\right)e^{-ik_{x}x},$$

where we have defined

(26) $$\begin{array}{cc} e^{i\theta } & =\left(k_{x}+ik_{y}\right)/k_{F},\\ k_{F} & =\left(E-V(x)\right)/\hbar v_{F},\\ \mathrm{and}\;\;\;\;k_{x} & =\sqrt{k_{F}^{2}-k_{y}^{2}}. \end{array}$$

For E<V(x), propagating wave solutions also exist (this is, by the way, the main difference between the solutions of the massless Dirac equation and the Schrödinger equation, which leads, in particular, to the phenomenon known as Klein tunneling [65,66]) and differ from those given in Equation (25) only in some signs. We leave the straightforward derivation to the reader.

At the interface of regions differing in the (locally constant) value of V(x), we perform a matching of wave functions, which for the two-dimensional Dirac equation reduces to solving the continuity conditions for both spinor components (Similar to the Schrödinger equation case, the wave function matching conditions are a consequence of charge conservation. To obtain the current density operator, in the Hamiltonian operator on the left side of Equation (20), $H=v_{F}\;p\cdot \sigma +V\left(x\right)$, we substitute p→p+eA, and then differentiate the resulting operator H(A) with respect to the vector potential A. In effect, we obtain $j=ev_{F}\sigma$, and therefore the continuity of the current corresponds to the continuity of the components of the wave function (operator j does not involve differentiation over coordinates)). For instance, if we consider the scattering from the right side of the discontinuity to the left side, we write

(27) $$t\phi^{\left(L,-\right)}=\phi^{\left(R,-\right)}+r\phi^{\left(R,+\right)},$$

where the spinor functions with indices *L* and *R* differ in the values of $k_{F}$ and $k_{x}$ [see Equations (25) and (26)], but are characterized by the same value of $k_{y}$. Since the considerations concern the interface between the graphene sample and the graphene region covered with a metal electrode (see Figure 3a), the calculations can be simplified by adopting the model of a heavily doped electrode, in which we set $V\left(x\right)=-V_{\infty }$, where $V_{\infty }\to \infty$; we can then write the wave functions on the left in asymptotic form

(28) $$\phi^{\left(L,\pm \right)}≃\left(\begin{array}{c} 1\\ \pm 1 \end{array}\right),$$

where we have omitted the phase factor, which is not important for further considerations. After substituting the above into Equation (27), the calculations are straightforward; we now present the results for the transition and reflection probabilities

(29) $$T_{1}={\left|t\right|}^{2}=\frac{2\cos\theta }{1+\cos\theta },\;\;\;\;R_{1}={\left|t\right|}^{2}=\frac{1-\cos\theta }{1+\cos\theta },$$

which turn out to depend only on the angle of incidence θ of the plane wave, or—more precisely—on the value of $\cos\theta =\sqrt{1-{\left(k_{y}/k_{F}\right)}^{2}}$. In particular, we see that for θ=0 we have $T_{1}=1$ (and $R_{1}=0$), which is a manifestation of the Klein tunneling mentioned above (let us emphasize that the potential barrier considered here has an infinite height).

The probability of passing through the entire graphene sample, i.e., through two electrostatic potential steps occurring at the sample–lead interface (see Figure 3a), is most easily calculated using the double-barrier formula, the clear derivation of which can be found, e.g., in the Datta’s handbook [67]

(30) $$T_{12}=\frac{T_{1}T_{2}}{1+R_{1}R_{2}-2\sqrt{R_{1}R_{2}}\cos\phi },$$

where a phase shift

(31) $$\phi =k_{x}L=L\sqrt{k_{F}^{2}-k_{y}^{2}},$$

related to the propagation of a plane wave along the main axis *x* is introduced (note here that the phase shift introduced in this manner also implies the assumption that any reflections from the side edges of the system do not change the value of $k_{y}$; in practice, this implies that we restrict our considerations to systems for which W≫L). Assuming barrier symmetry, $T_{2}=T_{1}$, $R_{2}=R_{1}$, and substituting the formulas given in Equation (29), we can now write $T_{12}$ explicitly as a function of $k_{y}$ and *E*

(32) $$T_{12}=T_{k_{y}}\left(E\right)={\left[1+{\left(\frac{k_{y}}{\varkappa }\right)}^{2}\sin^{2}\left(\varkappa \;L\right)\right]}^{-1},$$

where

(33) $$\varkappa =\left\{\begin{array}{cc} \sqrt{k_{F}^{2}-k_{y}^{2}}, & \mathrm{for}\;\;|k_{y}|⩽k_{F},\\ i\sqrt{k_{y}^{2}-k_{F}^{2}}, & \mathrm{for}\;\;|k_{y}|>k_{F}, \end{array}\right.$$

and the Fermi wave vector, assuming V(x)=0 for the sample region, is equal to $k_{F}=\left|E\right|/\left(\hbar v_{F}\right)$. The absolute value in the last expression arises from the fact that formulas in Equation (29) and the following results are identical for E<0; we leave the verification of this property to the reader.

### 2.5. The Conductivity, Shot Noise, and Higher Cumulants for Ballistic Graphene Strip

The physical consequences of the above expression for the transition probabilities $T_{k_{y}}\left(E\right)$, see Equations (32) and (33), are now discussed for two physical situations: a charge-neutral sample ($k_{F}=0$) and the Sharvin limit ($k_{F}W\gg 1$) (unless otherwise stated, we also assume geometry with long, parallel sample–lead interfaces and W≫L).

In the first case ($k_{F}=0$), we obtain $\varkappa =i|k_{y}|$ and can use the identity sin(ix)=isinhx, resulting in a surprisingly simple expression

(34) $$T_{k_{y}}\left(0\right)=\frac{1}{\cosh^{2}\left(k_{y}L\right)},$$

visualized in Figure 3b. In the wide-sample limit, W≫L, the sums appearing in the formulas for Landauer conductance *G* [see Equation (5)], Fano factor *F* [Equation (12)], and higher cumulants $R_{3}$, $R_{4}$ [Equations (17) and (18)] can be approximated with integrals [see Equation (24)], leading to

(35) $$\begin{array}{cc} \sum_{n}T_{n}\left(0\right) & \approx \frac{W}{\pi }\int_{0}^{\infty }dk_{y}\;T_{k_{y}}\left(0\right)=\frac{W}{\pi L}, \end{array}$$

(36) $$\begin{array}{cc} \sum_{n}{\left[T_{n}\left(0\right)\right]}^{2} & \approx \frac{W}{\pi }\int_{0}^{\infty }dk_{y}\;{\left(T_{k_{y}}\left(0\right)\right)}^{2}=\frac{2}{3}\frac{W}{\pi L}, \end{array}$$

or, more generally,

(37) $$\sum_{n}{\left[T_{n}\left(0\right)\right]}^{m}\approx \frac{W}{2\sqrt{\pi }\;L}\;\frac{\Gamma (m)}{\Gamma (m+1/2)}\;\;\;\;\mathrm{for}\;\;m>0,$$

where Γ(x) is the Euler gamma function. To facilitate future comparisons with other transport regimes, we will additionally define

(38) $${\langle T^{m}\rangle }_{k_{F}=0}=L\int_{0}^{\infty }dk_{y}\;{\left(T_{k_{y}}\left(0\right)\right)}^{m}=\frac{\sqrt{\pi }\;\Gamma \left(m\right)}{2\Gamma (m+\frac{1}{2})},$$

such that ${\langle T\rangle }_{k_{F}=0}=1$. Taking into account the graphene-specific fourfold degeneracy of states due to the presence of spin and valley degrees of freedom (the conductance quantum is therefore $g_{0}=4e^{2}/h$), we obtain

(39) $$\begin{array}{c} G\approx \frac{4e^{2}}{\pi h}\frac{W}{L},\;\;\;\;F\approx 1-\frac{2}{3}=\frac{1}{3}, \end{array}$$

(40) $$\begin{array}{c} R_{3}\approx \frac{1}{15},\;\;\;\;R_{4}\approx -\frac{5}{512}. \end{array}$$

The value of G∝W/L (instead of G∝W, as in a typical ballistic system) means that charge-neutral graphene exhibits universal specific conductivity, $\sigma_{0}=4e^{2}/\left(\pi h\right)$, the value of which is additionally determined only by the universal constants of nature. The value of the Fano factor F=1/3 is also not accidental, as it is a value characteristic for ohmic (disordered) conductors (the same applies to higher cumulants). In the context of graphene, the term *pseudodiffusive conductivity* is often used to emphasize that this ballistic system perfectly emulates an ohmic conductor within the appropriate parameter range. It should be emphasized that the first two theoretical values, given in Equation (39) and originally derived in Ref. [14] (conductance) and [18] (Fano factor), have been experimentally confirmed with satisfactory accuracy in 2008 [19] (for the comprehensive theoretical discussion of full counting statistics for graphene at the Dirac point, see Ref. [68]).

In the Sharvin limit ($k_{F}W\gg 1$), the situation looks slightly different. We can then assume that the contribution of modes for which $k_{y}>k_{F}$ (i.e., *evanescent* modes) is negligible and limits the considerations to $k_{y}⩽k_{F}$. Next, we notice that as the values of $T_{k_{y}}\left(E\right)$ (or their powers) are summed, the $\sin^{2}\left(L\sqrt{k_{F}^{2}-k_{y}^{2}}\right)$ term of Equation (32) oscillates rapidly, especially as $k_{y}$ approaches $k_{F}$. Therefore, it seems reasonable to replace the sine argument with a random phase and average the result, which leads to the following approximation

(41) $$\begin{array}{cc} T_{k_{y}}\left(E\right)\approx {\left\{T_{k_{y}}\right\}}_{\mathrm{incoh}} & =\frac{1}{\pi }\int_{0}^{\pi }\frac{d\varphi }{1+\left(k_{y}^{2}/\varkappa^{2}\right)\sin^{2}\varphi }\\ \; & =\sqrt{1-{\left(k_{y}/k_{F}\right)}^{2}}, \end{array}$$

where we used the table integral [69]

(42) $$I\left(a,b\right)=\frac{1}{2\pi }\int_{-\pi }^{\pi }\frac{du}{a+b\cos u}=\frac{1}{\sqrt{a^{2}-b^{2}}},\;\;\;\;\mathrm{for}\;a>\left|b\right|,$$

substituting

(43) $$\begin{array}{cc} u & =2\varphi ,\\ a & =\frac{1-\frac{1}{2}\eta^{2}}{1-\eta^{2}},\;\;\;\;b=a-1=\frac{\frac{1}{2}\eta^{2}}{1-\eta^{2}},\\ \mathrm{with}\;\;\eta & =k_{y}/k_{F}. \end{array}$$

The comparison between the approximation given in Equation (41) and the actual $T_{k_{y}}\left(E\right)$—see Equations (32) and (33)—for $k_{F}L=25$ is presented in Figure 3d.

Equation (41) is essentially the Dirac version of Equation (7) describing a standard ballistic system; when calculating the Landauer conductance, we can again approximate the summation by integration and obtain

(44) $$G\approx \frac{g_{0}W}{\pi }\int_{0}^{k_{F}}dk_{y}\;{\left\{T_{k_{y}}\right\}}_{\mathrm{incoh}}=\frac{\pi }{4}G_{\mathrm{Sharvin}},$$

where we recall the value of Sharvin conductance given in Equation (8). The prefactor π/4 is a consequence of the fact that in the last expression in Equation (41), where previously there was a step function $\Theta (k_{F}-k_{y})$, a term describing an arc of a circle appeared; the conductance of graphene beyond the charge neutrality point is therefore reduced compared to a typical ballistic system.

Interestingly, in deriving Equation (44), we did not explicitly assume, as in Equation (39), that the width to length ratio of the sample is W/L≫1; hypothetically, the result given in Equation (44) can therefore be applied whenever the condition $k_{F}W\gg 1$ is satisfied, regardless of the value of W/L. In practice, however, it is difficult to imagine that the double-barrier transmission formula, Equation (30), which is the basis of the entire reasoning, could be applied to samples that do not satisfy the W/L≫1 condition. It seems that in the case of graphene samples with L≳W, hard-to-control edge effects can significantly alter the conductivity [70]. We will address this issue later in this paper, but first let us calculate the approximate values of the Fano factor and higher cumulants in the Sharvin limit.

In order to construct the approximation analogous to this in Equation (41) but for $T_{k_{y}}^{2}$, it is sufficient to calculate

(45) $$\begin{array}{cc} {\left\{T_{k_{y}}^{2}\right\}}_{\mathrm{incoh}} & =\frac{1}{\pi }\int_{0}^{\pi }\frac{d\varphi }{{\left[1+\left(k_{y}^{2}/\varkappa^{2}\right)\sin^{2}\varphi \right]}^{2}}\\ \; & =\sqrt{1-\eta^{2}}\left[1-\frac{1}{2}\eta^{2}\right], \end{array}$$

where we used the first derivative of I(a,b)—see Equations (42) and (43)—with respect to *a*. More generally, the *m*-th power of $T_{k_{y}}$ can be approximated by

(46) {Tkym}incoh=1π∫0πdφ1+ky2/ϰ2sin2φm=(−1)m−1(m−1)!∂m−1∂am−1I(a,b)=2m−1Γ(m−12)am−1πΓ(m)(a2−b2)m−1/2F121−m2,1−m2;32−m;1−b2a2,=2m−1Γ(m−12)πΓ(m)1−η2z−m+1F121−m2,1−m2;32−m;z,withz=1−η2(1−12η2)2,

where F12(α,β;γ;z) is the hypergeometric function [71] (notice that for a positive integer *m*, $\alpha =1-\frac{m}{2}$ or $\beta =\frac{1-m}{2}$ is a non-positive integer, so the function reduces to a polynomial of *z*; after multiplying by $z^{-m+1}$, the resulting expression can be further simplified to a degree 2m−2 polynomial of the variable η). For m=1 and m=2, the above reproduces the results of Equations (41) and (45), respectively. The next two expressions are

(47) $$\begin{array}{cc} {\left\{T_{k_{y}}^{3}\right\}}_{\mathrm{incoh}} & =\sqrt{1-\eta^{2}}\left(1-\eta^{2}+\frac{3}{8}\eta^{4}\right), \end{array}$$

(48) $$\begin{array}{cc} {\left\{T_{k_{y}}^{4}\right\}}_{\mathrm{incoh}} & =\sqrt{1-\eta^{2}}\left(1-\frac{\eta^{2}}{2}\right)\left(1-\eta^{2}+\frac{5}{8}\eta^{4}\right). \end{array}$$

Setting ${\left[T_{k_{y}}\left(E\right)\right]}^{m}\approx {\left\{T_{k_{y}}^{m}\right\}}_{\mathrm{incoh}}$ for m=1,…,4, and approximating the summations occuring in Equations (12), (17), and (18) by integrations, namely,

(49) $$\begin{array}{cc} \sum_{n}{\left[T_{n}\left(E\right)\right]}^{m} & \approx \frac{k_{F}W}{\pi }\langle {\left\{T_{k_{y}}^{m}\right\}}_{\mathrm{incoh}}\rangle \end{array}$$

(50) $$\begin{array}{cc} \mathrm{with}\;\;\langle {\left\{T_{k_{y}}^{m}\right\}}_{\mathrm{incoh}}\rangle \; & =\int_{0}^{1}d\eta \;{\left\{T_{k_{y}}^{m}\right\}}_{\mathrm{incoh}}, \end{array}$$

we obtain

(51) $$F\approx \frac{1}{8},\;\;\;\;R_{3}\approx -\frac{1}{23},\;\;\;\;R_{4}\approx -\frac{5}{512}.$$

For the first four numerical values of $\langle {\left\{T_{k_{y}}^{m}\right\}}_{\mathrm{incoh}}\rangle$, see Table 1.

The surprising (nonzero) value of the shot noise in Equation (51) is close to the experimental results obtained by Danneau et al. [19], which are in the range of F=0.10÷0.15. (The aspect ratio of the sample used in this experiment was W/L=24.) Recently, for other 2D Dirac systems in the gapless limit, a theoretical prediction of F≈0.179 was reported [72]. It should be emphasized that measuring the shot noise of nanoscopic devices containing components of different materials is rather challenging; there are also results in the literature that suggest that the dependence of the shot noise on the system filling is weak, with the value always close to the pseudodiffusive F=1/3 [73]. Unlike for carbon nanotubes, where inelastic processes may lead to super-Poissonian noise with 1<F<3 [74], values of F>1 have not been detected for bulk graphene. The measurements of higher charge cumulants for graphene-based systems are so far missing.

**Figure 4:**
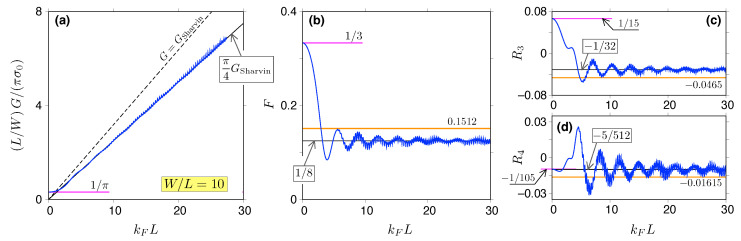
Conductance (**a**), Fano factor (**b**), third (**c**), and fourth (**d**) charge transfer cumulant for graphene strip dislayed as functions of the Fermi momentum (solid blue lines). The aspect ratio is fixed at W/L=10. The dashed black line in (**a**) depicts the Sharvin conductance $G_{\mathrm{Sharvin}}=g_{0}k_{F}W/\pi$, with $g_{0}=4e^{2}/h$; the sub-Sharvin values, given by Equations (44) and (51), are depicted with solid black lines in all panels. The short purple line (**a**–**d**) marks the pseudodiffusive value—see Equations (39) and (40)—that is approached for $k_{F}\to 0$. Wide orange lines (**b**–**d**) depict the values following from the approximated distribution of transmission probabilities $\rho_{\mathrm{approx}}\left(T\right)$; see Equation (61).

In Figure 4, the approximations for *G*, *F*, $R_{3}$, and $R_{4}$, both for $k_{F}=0$ and for $k_{F}W\gg 1$, are compared with and the actual values following from Equations (32) and (33) for $T_{k_{y}}\left(E\right)$. Briefly speaking, the higher the cumulant that is considered, the larger the value of $k_{F}W$ necessary to observe the convergence to the sub-Sharvin limit; however, for W/L=10 and for the heavily-doped leads, $k_{F}W≳5$ is sufficient. The discussion of more realistic situations (finite doping in the leads, other sample shapes) is presented later in this paper.

### 2.6. The Narrow Opening Limit

**Figure 5:**
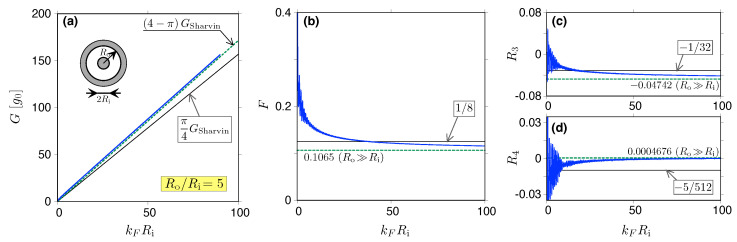
(**a**–**d**) Same as Figure 4 but for the Corbino disk; see inset in (**a**), with the outer-to-inner radii ratio $R_{o}/R_{i}=5$. Solid blue lines mark the exact results following from Equations (56) and (57). The remaining lines mark the sub-Sharvin values relevant for the thin-disk limit $R_{i}/R_{o}\to 1$ [solid black] and for the narrow opening limit, $R_{i}/R_{o}\to 0$; see Equation (55) [dashed green].

Using the conformal mapping technique [20,75], it can be shown that for charge-neutral graphene ($k_{F}=0$), the pseudodiffusive values given in Equations (37), (39), and (40) are essentially valid for an arbitrary sample shape, provided that the prefector W/L (if present) is replaced by an appropriate geometry-dependent factor defined by the conformal transformation. In particular, when mapping the rectangle onto the Corbino disk, one needs to substitute $W/L\to 2\pi /\log(R_{o}/R_{i})$, where $R_{o}$ and $R_{i}$ are the outer and inner disk radii (respectively); see Figure 5a. An additional condition is that the system must be in the multimode regime, i.e., $\log(R_{o}/R_{i})\ll 1$ (or $R_{i}\approx R_{i}$) in the disk case. Otherwise, if $R_{i}\ll R_{o}$, a nonstandard tunneling behavior is observed, with $G\propto R_{i}/R_{o}$ and F→1 [20] (since the transport is governed by a single mode, we also have $R_{3}\to 1$ and $R_{4}\to 1$ in such a case).

However, in the Sharvin limit ($k_{F}R_{i}\gg 1$ in the disk case), a different set of universal charge-transport characteristics is predicted [34]. Regardless of the exact size or shape of the outer sample–lead interface, one can assume that the double-barrier formula, Equation (30), is still applicable and that $T_{2}\approx 1$ and $R_{2}=1-T_{2}\approx 0$ due to the Klein tunneling. Therefore, $T_{12}\approx T_{1}$, the role of a phase shift ϕ is negligible, and one can write—for the wave leaving the inner lead with the total angular momentum ℏj (with j=±1/2,±3/2,…)—the transmission probability as

(52) $$\begin{array}{cc} {\left(T_{j}\right)}_{R_{i}\ll R_{o}} & \approx T\left(u_{j}\right)=\left\{\begin{array}{cc} \frac{2\sqrt{1-u_{j}^{2}}}{1+\sqrt{1-u_{j}^{2}}}\;\;\; & \mathrm{if}\;\;|u_{j}|\leq 1,\\ 0\;\; & \mathrm{if}\;\;|u_{j}|>1, \end{array}\right.\\ \mathrm{with}\;\;u_{j} & =\frac{j}{k_{F}R_{i}}. \end{array}$$

Subsequently, the summation for the *m*-th power of $T_{j}$ can be approximated (notice that we have assumed $k_{F}R_{i}\gg 1$) by integration over −1⩽u⩽1, leading to

(53) $$\sum_{j}{\left(T_{j}\right)}^{m}\approx k_{F}R_{i}\int_{-1}^{1}du{\left[T(u)\right]}^{m}=2k_{F}R_{i}{\langle T^{m}\rangle }_{u},$$

where, by also using the symmetry T(−u)=T(u), we have defined

(54) 〈Tm〉u=∫01du21−u21+1−u2m=πΓ(m+2)4Γ(m+52)2m+3−2mF1212,1;m+52;−1.

The first four values of ${\langle T^{m}\rangle }_{u}$ are listed in Table 1. Substituting the above into Equations (5), (12), (17), and (18), we obtain

(55) $$\begin{array}{ccc} G & \approx \left(4-\pi \right)\;G_{\mathrm{Sharvin}},\\ \; & \;\mathrm{with}\;\;G_{\mathrm{Sharvin}}=2g_{0}k_{F}R_{i},\\ F & \approx \frac{9\pi -28}{3(4-\pi )}≃0.1065,\; & \left(R_{i}\ll R_{o}\right)\\ R_{3} & \approx \frac{204-65\pi }{5(4-\pi )}≃-0.04742,\\ R_{4} & \approx \frac{1575\pi -4948}{21(4-\pi )}≃0.0004674. \end{array}$$

For the Corbino disk, it is also possible to perform the analytical mode matching for the heavily-doped leads, but also for the arbitrary disk doping $k_{F}$ and radii ratio $R_{o}/R_{i}$ [20,76]. We skip the details of the derivation here and just give the transmission probabilities

(56) $$T_{j}=\frac{16}{\pi^{2}k^{2}R_{i}R_{o}}\;\frac{1}{{\left[D_{j}^{\left(+\right)}\right]}^{2}+{\left[D_{j}^{\left(-\right)}\right]}^{2}},$$

where

(57) Dj(±)=ImHj−1/2(1)(kFRi)Hj∓1/2(2)(kFRo)±Hj+1/2(1)(kFRi)Hj±1/2(2)(kFRo),

and $H_{\nu }^{\left(1\right)}\left(\rho \right)$ [$H_{\nu }^{\left(2\right)}\left(\rho \right)$] is the Hankel function of the first [second] kind. In Figure 5, we provide the comparison between the values of the conductance and the next three charge cumulants obtained by substituting the exact $T_{j}$-s given above into Equations (5), (12), (17), and (18), and perform the numerical summation over *j* with the approximate values given in Equation (55). It is easy to see that the radii ratio of $R_{o}/R_{i}=5$ is sufficient to reproduce our predictions for the narrow opening limit, with good accuracy, typically starting from $k_{F}R_{i}=50-100$ (this time, the higher cumulant is considered, so there is faster convergence).

## 3. Distributions of Transmission Probabilities

A compact and intuitive representation of the charge transfer cumulants discussed in the previous Section is provided within the distribution function of transmission probabilities ρ(T). This function takes the simplest form when the transmission probability can be expressed as a monotonic function of the parameter λ, i.e., T=T(λ). In such a case, the probability density is defined by

(58) $$\rho \left(T\right)=\rho \left(\lambda \right)\left|\frac{d\lambda }{dT}\right|,$$

where ρ(λ) is the number of transmission channels per unit of λ (here, this is constant and determined by the appropriate quantization rule, see Equations (6), (24), or (52)), and dλ/dT is the derivative of the inverse function λ(T). In a generic situation, the right-hand side in Equation (58) needs to be replaced by the sum over the monotonicity intervals of T=T(λ).

In the pseudodiffusive limit, $k_{F}=0$ and W≫L, the transmission probability given by Equation (34) immediately implies

(59) $$\rho_{\mathrm{diff}}\left(T\right)=\frac{W}{2\pi L}\;\frac{1}{T\sqrt{1-T}}=\frac{G_{\mathrm{diff}}}{2\pi \sigma_{0}}\;\frac{1}{T\sqrt{1-T}},$$

where we recall the pseudodiffisive conductance $G=G_{\mathrm{diff}}$ given in Equation (39). The distribution $\rho_{\mathrm{diff}}\left(T\right)$ is visualized in Figure 3c.

Analyzing the sub-Sharvin transport, we now change the order of presentation by switching to the disk geometry to point out that in the narrow opening limit, i.e., for $k_{F}R_{i}\gg 1$ and $R_{i}\ll R_{o}$, the transmission probability $T(u_{j})$ given by Equation (52) leads to another closed-form expression for the distribution, namely

(60) $$\rho_{R_{i}\ll R_{o}}\left(T\right)=\frac{G_{\mathrm{Sharvin}}}{g_{0}}\;\frac{T}{{\left(2-T\right)}^{2}\sqrt{1-T}},$$

with $G_{\mathrm{Sharvin}}=2g_{0}k_{F}R_{i}$ for a circular lead.

In the case of parallel interfaces at a distance *L*—see Figure 3a—the description of the sub-Sharvin transport becomes more complex, since the transmission probability $T_{k_{y}}\left(E\right)$—see Equations (32) and (33)—is no longer a monotonic function of $k_{y}$. The distribution ρ(T) obtained numerically for $k_{F}L=25$ is presented in Figure 3e, where the continuous $k_{y}$ corresponds to the W≫L limit. It can be seen that each of the seven transmission minima [see Figure 3d] produces a distinct (integrable) singularity of ρ(T) located at $0<T_{\min}<1$. A closed-form, asymptotic expression for ρ(T) in the $k_{F}\to \infty$ limit is missing; instead, we propose the approximation directly following from ${\left\{T_{k_{y}}\right\}}_{\mathrm{incoh}}$ given by Equation (41), i.e.,

(61) $$\rho_{\mathrm{approx}}\left(T\right)=\frac{G_{\mathrm{Sharvin}}}{g_{0}}\;\frac{T}{\sqrt{1-T^{2}}}.$$

Subsequent approximations for the cumulants can be evaluated as

(62) $${\langle T^{m}\rangle }_{\rho_{\mathrm{approx}}\left(T\right)}=\frac{g_{0}}{G_{\mathrm{Sharvin}}}\int_{0}^{1}dT\;T^{m}\rho_{\mathrm{approx}}\left(T\right),\;\;\;\;m⩾1.$$

The numerical values for m=1,…,2 are listed in Table 1, together with the corresponding approximations for the charge transfer cumulants *F*, $R_{3}$, and $R_{4}$, which are also depicted in Figure 4b–d (thick horizontal lines). We notice that these values typically match the incoherent ones, obtained by substituting Equation (46) into Equation (50), within the accuracy that allows the unambiguous identification of the transport regime. A surprising exception is the case of $R_{4}$, for which the proximity of the pseudodiffusive (−1/105) and incoherent (−5/512) values is merely a coincidence. (By definition, the conductance $G\approx \left(\pi /4\right)\;G_{\mathrm{Sharvin}}=\left(k_{F}W/\pi \right){\langle T\rangle }_{\rho_{\mathrm{approx}}\left(T\right)}$.)

The functional forms of ρ(T) derived in this Section, along with a selection of others previously reported in the literature, can be found in Table 2.

## 4. Numerical Results and Discussion

In this Section, we compare the predictions from analytical theory for the charge transfer cumulants (see Section 2) with the results of the computer simulations of electron transport in selected nanostructures in graphene shown in Figure 1 (for the parameters, see Table 3). The signatures of the sub-Sharvin transport regime originate from the multiple scattering between interfaces separating weakly and heavily doped regions; therefore, it is important to compare the results for different crystallographic orientations of such interfaces. We note that for the system of Figure 1a, the interfaces are parallel to the armchair direction, for the system of Figure 1c, the semicircular interfaces probe all crystallographic orientations, while for the systems of Figure 1d,e, the interfaces are parallel to the zigzag direction.

Since discrete structures carved out of a honeycomb lattice exhibit Fabry–Pérot-type oscillations in all studied transport properties as a function of Fermi energy, and (typically) the higher the cumulant, the larger the oscillation magnitude, we limit the forthcoming discussion to the Landauer–Büttiker conductance (*G*) and the Fano factor (*F*). It is also worth noting that the ratio of the former to the Sharvin conductance ($G/G_{\mathrm{Sharvin}}$) accompanied by *F* provides sufficient information to unambiguously identify one of the basic quantum transport regimes if applicable; see Table 2.

### 4.1. Tight Binding Model

This part of the analysis starts from the tight-binding model of graphene, with Hamiltonian

(63) $$H=\sum_{i,j,s}t_{ij}c_{i,s}^{†}c_{j,s}+\sum_{i,s}V_{i}n_{is},$$

where the indices *i*, *j* run over sites in the honeycomb lattice of carbon atoms, and s=↑,↓ is the spin up/down orientation. The hopping-matrix elements are given by

(64) $$t_{ij}=\left\{\begin{array}{cc} -t_{0} & \mathrm{if}\;i,j\;\mathrm{are}\;\mathrm{nearest}-\mathrm{neighbors},\\ 0 & \mathrm{otherwise}, \end{array}\right.$$

with $t_{0}=2.7\;$eV. For the systems shown in Figure 1a,d,e, the electrostatic potential energy $V_{j}=V\left(x_{j}\right)$ varies only along the main axis. It equals $-V_{\mathrm{infty}}$, with $V_{\mathrm{infty}}=t_{0}/2=1.35\;$eV in the leads which is raised to $V_{j}=0$ in the sample area. The abrupt potential increase at the sample–lead interface is smoothed over the length $L_{s}$, according to the function

(65) $$\Theta_{L_{s}}\left(x\right)=\left\{\begin{array}{cc} 0 & \mathrm{if}\;\;x<-L_{s}/2,\\ \frac{1}{2}+\frac{1}{2}\sin\left(\pi x/L_{s}\right)\; & \mathrm{if}\;\;|x|⩽L_{s}/2,\\ 1 & \mathrm{if}\;\;x>L_{s}/2. \end{array}\right.$$

The potential barrier, composed of two steps at $x=x_{1}$ and $x=x_{2}\equiv x_{1}+L$, namely

(66) $$V\left(x\right)=V_{\infty }\left[\;\Theta_{L_{s}}\left(x-x_{1}\right)-\Theta_{L_{s}}\left(x-x_{2}\right)\;\right]-V_{\infty },$$

is rectangular for $L_{s}=0$ [solid line in Figure 1b], whereas it has a sinusoidal shape for $L_{s}=L$ [dashed line]. For the half-disk shown in Figure 1c, we simply take $V_{j}=V\left(r_{j}\right)$, where $r_{j}$ is the radius in polar coordinates, with the same function V(r) as in Equation (66). The interface positions ($x_{1},x_{2}$) coincide with the ends of the central (narrowest) part with parallel edges (see Figure 1a), the inner/outer disk radii (Figure 1c), or with the neckings limiting the dot region (Figure 1d,e). The remaining symbols in Equation (63) are a creation (annihilation) operator for an electron with spin *s* at lattice site *i*, $c_{i,s}^{†}$ ($c_{i,s}$), and the particle number operator $n_{is}=c_{i,s}^{†}c_{i,s}$ (since the Hamiltonian (63) can be represented as the sum of the two commuting terms, one for s=↑ and the other for s=↓, it is sufficient to calculate the transport characteristics for one spin direction and to multiply the results by the degeneracy factor 2).

In the following section, we consider $0⩽L_{s}⩽L$ only for the constriction shown in Figure 1a; once the effect of the smooth potential barrier is identified, the discussion of the remaining systems concentrates on the case of $L_{s}=0$ (i.e., abrupt step).

Although electron–electron interaction is neglected in the tight-binding model, Equation (66) with $L_{s}>0$ can be regarded as a simplified description of carrier diffusion in a real device, leading to a smooth variation in the effective (self-consistent) potential in an interface between regions of different doping [80,81]. The possible role of electron–electron interactions in graphene has been discussed by numerous authors [82,83,84,85,86,87,88], leading to the conclusion that the correlation effects (not tractable within the mean-field description) are typically negligible, and—unless extreme stains are applied [86,87]—may manifest themselves only via partial magnetic order at the sample edges [88].

The nearest-neighbor hopping Hamiltonian (64) already grasps several features of more accurate models, such as the trigonal warping of the dispersion relation and the presence of the van Hove singularity in the density of states [89,90]. For a discussion of Hamiltonians with more distant hopping elements, see Ref. [91].

### 4.2. Constriction with Zigzag Edges

As a first example of the system, for which the analytical mode matching technique presented in Section 2 cannot be directly applied, we consider the constriction with zigzag edges, earlier considered as the valley [92] or spin [93,94] filter, depicted in Figure 1a. The central section of this system is an almost perfect square, with the length L=104a≃25.58 nm and the width $W=60\sqrt{3}\;a≃25.57\;$ nm (see also Table 3) attached to wedge-shaped electrodes that evolve into wide stripes with the width $W_{\mathrm{infty}}=210\sqrt{3}\;a≃89.5\;$nm. Such a geometry is chosen to mimic the typical experimental situation, in which the nanostructure in graphene is contacted by much wider metallic leads [95]. Also, the potential step height, $V_{\infty }=t_{0}/2≃1.35\;$eV, is not far from the results of some first-principles calculations for graphene–metal structures [80,81]. Semi-infinite leads of a constant width $W_{\infty }$ play the role of the waveguides, as shown in Figure 2; they can be divided into the repeating supercells in order to find the propagating modes numerically by adapting the scheme developed by Ando for a square lattice [96] to the honeycomb lattice. For the potential profile given by Equations (65) and (66), the number of propagating modes (per one direction) is equal in the left and right leads, $N_{L}=N_{R}$ (It can be further approximated as $N_{\mathrm{approx}}=2KW_{\mathrm{infty}}/\pi$ with $K=|E+V_{\infty }|/\hbar v_{F}$, giving $N_{\mathrm{approx}}≃130$ for E=0 and $V_{\infty }=t_{0}/2$; the actual number of propagating modes $N_{\mathrm{open}}^{\left(\infty \right)}⩾N_{\mathrm{approx}}$ (if $|E+V_{\infty }|≲t_{0}$) due to the trigonal warping).

Since the central section of the system is bounded by two parallel interfaces separating weakly and heavily doped regions, one can expect that the key findings for a graphene strip in the sub-Sharvin regime—see Equations (44) and (51)—still apply, at least for $L_{s}\ll L$. However, the system width now varies with the position along the main axis, so the scattering cannot be described independently for each normal mode, as in Equation (27). Instead, the mode mixing occurs, and—if scattering from the left is considered—we can define the transmission matrix $t=(t_{mn})$, with $m=1,\ldots ,N_{R}$ and $n=1,\ldots ,N_{L}$, and the reflection matrix $r=(r_{mn})$, with $m=1,\ldots ,N_{L}$, $n=1,\ldots ,N_{L}$. The details of the calculations are presented in Appendix A; here we only mention that Equations (5) and (12) for measurable quantities remain valid, provided that the transmission probabilities $T_{n}$ are defined as eigenvalues of the matrix $tt^{†}$. Alternatively, one can express the Landauer–Büttiker conductance and the Fano factor in the basis-independent form, referring to the traces of the matrices $tt^{†}$ and ${\left(tt^{†}\right)}^{2}$, namely

(67) $$\begin{array}{cc} G & =\frac{2e^{2}}{h}\;Tr\left(tt^{†}\right), \end{array}$$

(68) $$\begin{array}{cc} F & =1-\frac{Tr{\left(tt^{†}\right)}^{2}}{Tr\left(tt^{†}\right)}. \end{array}$$

The factor 2 in Equation (67) denotes the spin degeneracy. The valley degeneracy of the transmission eigenvalues is now only approximate, since the dispersion relation following from the Hamiltonian (63) is no longer perfectly conical, but shows the trigonal warping [15] (for zigzag edges and electron doping, exact valley degeneracy occurs for all but one mode; for armchair edges, the degeneracy is approximate for all modes [97,98]).

**Figure 6:**
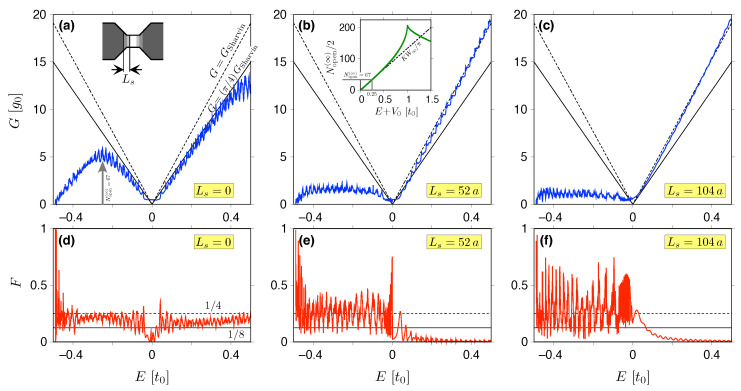
Conductance in the units of $g_{0}=4e^{2}/h$ (**a**–**c**) and the Fano factor (**d**–**f**) for the constriction with zigzag edges—see Figure 1a—displayed as functions of the Fermi energy defined with respect to the top of the electrostatic potential barrier in the narrow region—see also Figure 1b. The subsequent panels correspond to abrupt ($L_{s}=0$), partly-smooth ($L_{s}=L/2=52\;a$), and fully-smooth ($L_{s}=L=104\;a$) potential steps. The numerical results following from the tight-binding calculations are depicted with thick lines. Thin solid lines mark the sub-Sharvin values given by Equations (44) and (51); dashed lines in (**a**–**c**) mark the Sharvin conductance given by Equation (8) or, in (**d**–**f**), the shot noise power characterizing symmetric cavity, F=1/4 (the constriction width is $W=60\sqrt{3}\;a$; for the remaining simulation details, see Table 3.) The inset in (**b**) presents the number of propagating modes in the leads versus the energy $E+V_{0}$, with the step height $V_{0}=t_{0}/2=1.35\;$eV.

The results of our computer simulations are depicted by the thick colored lines in Figure 6. They match the sub-Sharvin values (marked by black solid lines) for electron doping (E>0) and the abrupt potential step ($L_{s}=0$). For hole doping (E<0) and $L_{s}=0$, the conductance *G* is still close to $\left(\pi /4\right)\;G_{\mathrm{Sharvin}}$ as long as the number of propagating modes in the leads is sufficiently large (see the inset in Figure 6b). At the same time, the Fano factor is rather closer to the value of F=1/4, which characterizes the symmetric cavity. In contrast, for smooth bariers ($L_{s}\gg a$), we have $G\approx G_{\mathrm{Sharvin}}$ for E>0 and $G\ll G_{\mathrm{Sharvin}}$ for E<0 (the conductance suppression due to the presence of two p-n junctions), as can be expected for the standard (i.e., Schödinger) ballistic system. At the same time, the Fano factor switches from F≪1 (for E>0) to F≈1/4 (for E<0). These findings are consistent with the results for smooth potential barriers and a strip with parallel edges, with mass confinement, presented in Ref. [33].

We see then that the constriction with zigzag edges carved out of a honeycomb lattice preserves all the key features of the idealized Dirac system studied previously.

### 4.3. Half-Disk and Circular Quantum Dots

**Figure 7:**
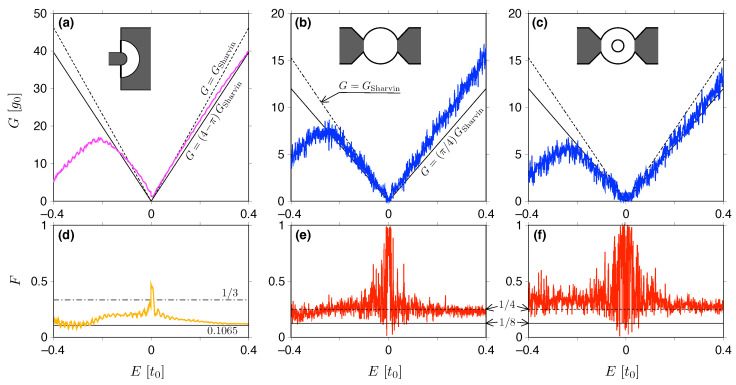
Same as in Figure 6, but for the half-Corbino disk (**a**,**d**) [see also Figure 1c], circular quantum dot (**b**,**e**) [see Figure 1d], and circular quantum dot with a circular hole (**c**,**f**) [see Figure 1e]. Thin solid lines in (**a**) and (**d**) show the results given in Equation (55) for the narrow opening limit; dash-dotted line in (**d**) marks the pseudodiffusive shot noise power, F=1/3. Remaining lines are same as in Figure 6. Other simulation details are given in Table 3.

We now focus on the case of the abrupt potential step ($L_{s}=0$) and consider the geometries for which the possible role of the edges is reduced (the half-Corbino disk) or amplified (circular quantum dot, without- or with a circular hole) compared to the constriction discussed above. It is worth mentioning here that previous numerical studies on similar nanostructures, either on the valley (or spin) filters [92,93,94] or the remaining systems [20,99,100], have focused on the few-mode energy range, making it difficult or impossible to distinguish between Sharvin and sub-Sharvin transport regimes (the same applies to the recent experimental study of graphene rings [101]). The conductance and the Fano factor determined from Equations (67) and (68) after the numerical calculation of the corresponding transmission matrix (see also Appendix A) are presented in Figure 7.

In the half-disk case—see Figure 7a,d—the conductance (for E>0) remains in the interval $G_{\mathrm{Sharvin}}≳G≳\left(4-\pi \right)\;G_{\mathrm{Sharvin}}$ (notice that the radii ratio is $R_{2}/R_{1}=4$, and thus the relevant analytic approximations are given in Equation (55) for the narrow opening limit), with a tendency to approach the narrow opening value with increasing *E*. For E<0, the conductance behavior is less clear, but the values of *G* are still close to both $G_{\mathrm{Sharvin}}$ and $\left(4-\pi \right)\;G_{\mathrm{Sharvin}}$. In contrast, the Fano factor is close to the narrow opening value of F≈0.1065 for both E>0 and E<0, except in the small vicinity of the charge neutrality point (E=0), where it is noticeably closer to the pseudodiffusive value of F=1/3.

For circular quantum dots, Fabry–Pérot interference combined with scattering from irregular sample edges, leads to much more pronounced oscillations of both *G* and *F*, discussed as functions of the Fermi energy, than in the case of a half-Corbino disk. In addition, the spectra presented in Figure 7b,c for *G* and Figure 7e,f for *F* suggest that the first charge transfer characteristic (*G*), discussed in isolation, may lead to the misidentification of the Sharvin or sub-Sharvin transport regime. Looking at the *F* spectra, it is clear that the chaotic cavity (with F=1/4) is the closest of the simple models that captures key features of the circular quantum dot (both in the variant without- or with a hole), at least for higher electron or hole dopings. The conductance itself, related to the Sharvin value for E>0, appears to be misleadingly close to $G_{\mathrm{Sharvin}}$ in the absence of a hole, or to $\left(\pi /4\right)\;G_{\mathrm{Sharvin}}$ in the presence of a hole (for E<0, the suppression of *G* due to p-n junctions occurs in both cases).

Therefore, complex nanostructures with irregular edges may accidentally show some features of Sharvin (or sub-Sharvin) transport, but the systematic discussion of quantum transport unveils the chaotic nature of the system.

## 5. Conclusions

The main purpose of the work was to better understand the novel *sub-Sharvin* transport regime in doped graphene, both by investigating the analytical solutions for idealized systems and by comparing the results for selected measurable quantities with computer simulations performed for more realistic nanostructures. For this goal, we have developed the analytical technique that allows one to calculate arbitrary charge transfer cumulant for doped graphene sample in two distinct physical situations: (i) two long and parallel abrupt interfaces separating the sample and the leads; (ii) a narrow circular interface governing transport through the much wider sample toward an external lead. In both cases, compact expressions are available for sufficiently high sample doping (infinite doping is assumed for the leads), for which multiple scattering between the interfaces can be taken into account, imposing the random phase each time the electron passes the sample area.

For the sake of completeness, we have also reviewed the most common quantum transport regimes described in the literature, with their statistical distributions of transmission probabilities. Evidence for a sub-Sharvin transport regime in doped graphene is pointed out.

Next, the results of analytical considerations for idealized systems are compared with computer simulations of quantum transport for more realistic systems carved out of a honeycomb lattice. The effects of finite doping in the leads, smooth potential steps, trigonal warping, and irregular sample edges are included in our simulations. The results show that the main features of the analytical approach discussed in the first part, which defines the sub-Sharvin transport regime in graphene (with its variants for parallel interfaces and for the narrow opening limit), are well reproduced in discrete systems on a honeycomb lattice, provided that the sample edges are straight and relatively short; i.e., with a total length comparable to or shorter than the total length of the sample–lead interfaces. In contrast, for systems with long and irregular edges, different charge transfer cumulants may suggest different quantum transport regimes, making unambiguous classification difficult or impossible.

Although our paper focuses on graphene, we expect that the main effects will also occur in other two-dimensional crystals such as silicene, germanene, or stanene [102,103]. This prediction is based on the nature of the results presented, in particular the fact that the occurrence of the sub-Sharvin transport regime is related to the conical dispersion relation rather than to the transmission via evanescent waves, which is responsible for the graphene-specific phenomena that occur at the charge neutrality point.

On the other hand, it seems unclear whether (or not) the sub-Sharvin transport regime could appear in systems showing interplay between the conical and quartic dispersion relations, such as bilayer graphene [104,105], mono-bilayer junctions [31], or hypothetical graphene–goldenene junctions [106]. These issues represent avenues for future theoretical and experimental research.

## Figures and Tables

**Table 1:** The first four cumulants for the transmission probabilities, $\langle T^{m}\rangle$, for different transport regimes in graphene and the corresponding values of the four charge transfer characteristics; see Equations (5), (12), (17), and (18).

	Transport Regime (or Approximation)			
Cumulant	*Pseudodiffusive, *	*Sub-Sharvin,*	${\langle \;X\;\rangle }_{\rho_{\mathrm{approx}}\left(T\right)}$,	*Narrow Opening,*
	$\;k_{F}\;=\;0,\;W\;\gg \;L$ ^*(a)*^	$\;k_{F}W\gg 1$ ^*(b)*^	see Equation (62) ^*(c)*^	$\;k_{F}R_{i}\;\gg \;1,\;R_{i}\;\ll \;R_{o}$ ^*(d)*^
〈T〉	1	π/4	π/4	4−π
$\langle T^{2}\rangle \;\;$	2/3	7π/32	2/3	40/3−4π
$\langle T^{3}\rangle \;\;$	8/15	51π/256	3π/16	192/5−12π
$\langle T^{4}\rangle \;\;$	16/35	759π/4096	8/15	$32\left(332/105-\pi \right)$
$G/G_{\mathrm{Sharvin}}$	*∞* ^*(e)*^	π/4	π/4	4−π
F	1/3	1/8	1−8/3π≃0.1512	(9π−28)/3(4−π)
$R_{3}\;\;$	1/15	−1/32	5/2−8/π≃−0.04648	(204−65π)/5(4−π)
$R_{4}\;\;$	−1/105	−5/512	10−472/15π≃−0.01615	(1575π−4948)/21(4−π)

Expressions for $\langle T^{m}\rangle$ with arbirary m⩾1 are given by ^*(a)*^ Equation (38); ^*(b)*^ Equations (46) and (50); ^*(c)*^ Equation (62); ^*(d)*^ Equation (54). ^*(e)*^ For $k_{F}=0$, $G=g_{0}W/\pi L$, with $g_{0}=4e^{2}/h$, coincides with $G_{\mathrm{Sharvin}}=0$.

**Table 2:** Basic quantum transport regimes in selected nanosystems characterized by the conductance (*G*), the Fano factor (*F*), and statistical distribution of transmission probabilities ρ(T). Other symbols are the Fermi wavenumber $k_{F}$, the conductance quantum $g_{0}=2e^{2}/h$ for two-dimensional electron gas (2DEG) or $4e^{2}/h$ for graphene, and the number of open channels $N_{\mathrm{open}}$.

Transport Regime	System	*G*	*F*	ρ(T)	Refs.
*Standard ballistic*	Sharvin contact in 2DEG, width *W*	$\;G_{\mathrm{Sharvin}}=g_{0}k_{F}W/\pi \;$	0	$\;N_{\mathrm{open}}\;\delta \left(1-T\right)\;$	[36,37]
*(Pseudo)-diffusive*	Diffusive conductor	$g_{0}\ll G\ll G_{\mathrm{Sharvin}}$	1/3	$\;\frac{G}{2g_{0}}\frac{1}{T\sqrt{1-T}}\;$	[77,78]
	Charge-neutral graphene sample (width *W*, length *L*)	$\sigma_{0}\frac{W}{L}$, $\;\sigma_{0}=\frac{4e^{2}}{\pi h}$	1/3	$\;\frac{G}{2\pi \sigma_{0}}\frac{1}{T\sqrt{1-T}}\;$	[18,19]
	Charge-neutral graphene disk (inner radii $R_{i}$, outer radii $R_{o}$)	$\frac{2\pi \sigma_{0}}{\ln\left(R_{o}/R_{i}\right)}$			[20]
*Sub-Sharvin*	Doped graphene sample (width *W*, length *L*)	$\frac{\pi }{4}\;G_{\mathrm{Sharvin}}$	1/8	$\approx \frac{G_{\mathrm{Sharvin}}}{g_{0}}\frac{T}{\sqrt{1-T^{2}}}\;$	[33], this work
	Doped graphene disk, B=0 (inner radii $R_{i}$, outer radii $R_{o}$)	$\;\frac{\pi }{4}$ ^*(a)*^ $<\frac{G}{{G_{\mathrm{Sharvin}}}^{\left(b\right)}}<4-\pi$ ^*(c)*^	1/8 ^*(a)*^ >F>0.1065 ^*(c)*^	$\frac{(G_{\mathrm{Sharvin}}/g_{0})\;T}{{\left(2-T\right)}^{2}\sqrt{1-T}}$ ^*(c)*^	[34], this work
	Doped graphene disk, $B\to B_{c,2}-$ ^*(d)*^	G→0	0.5497 ^*(a)*^ <F<1 ^*(c)*^	—	[40]
*Chaotic*	Symmetric cavity	$0<G<G_{\mathrm{Sharvin}}$ ^*(e)*^	1/4	$\frac{2G}{\pi g_{0}}\frac{1}{T^{1/2}\sqrt{1-T}}$	[79]

^*(a)*^ Reached for $R_{i}/R_{o}\to 1$. ^*(b)*^ Defined as $G_{\mathrm{Sharvin}}=2g_{0}k_{F}R_{i}$. ^*(c)*^ Reached for $R_{i}/R_{o}\to 0$. ^*(d)*^ Magnetic field corresponding to the vanishing conductance, $B_{c,2}=2\;\left(\hbar /e\right)k_{F}/\left(R_{o}-R_{i}\right)$. ^*(e)*^ Defined via the opening width *w*; i.e., $G_{\mathrm{Sharvin}}=g_{0}k_{F}w/\pi$.

**Table 3:** Detailed parameters of the systems studied numerically (see also Figure 1). For each case, the main spatial dimension is also given in physical units.

System	Defining Parameters	System (Sample) Length, $L_{\mathrm{tot}}\;\left(L^{\prime }\right)$ ^*(a)*^	No. of Sites ^*(b)*^
*Constriction with* *zigzag edges*	$W_{\infty }=210\sqrt{3}\;a$	254a≃62.5nm	105,452
	$\;W=60\sqrt{3}\;a,\;\;L=104\;a\;$	(≡L)	(24,960)
*Half-Corbino disk*	$W_{\infty }=700\;a$	$120\sqrt{3}\;a≃51.1\;$nm	336,000
	$R_{2}=4R_{1}=200\;a$	$\left(\;\equiv R_{2}\;-\;R_{1}\right)$	(136,035)
*Circular quantum dot*	$\;\;W_{\infty }\;=\;210\sqrt{3}\;a,\;\;R\;=\;105\sqrt{3}\;a\;$	512a≃126nm	320,881
	$w=60\sqrt{3}\;a$	(362a)	(240,389)
*Circular quantum dot* *with a circular hole*	$\;\;W_{\infty }\;=\;210\sqrt{3}\;a,\;\;R\;=\;105\sqrt{3}\;a\;$	512a≃126nm	301,148
	$w=60\sqrt{3}\;a,\;\;r=30\sqrt{3}\;a$	(362a)	(220,656)

^*(a)*^ $L_{\mathrm{tot}}$—the distance between semi-infinite leads; $L^{\prime }$—the distance between interfaces (given in parenthesis). ^*(b)*^ Total no. of sites between the leads (no. of sites with $V\left(x\right)>-V_{\infty }/2$ is given in parenthesis.)

**Figure A1:**
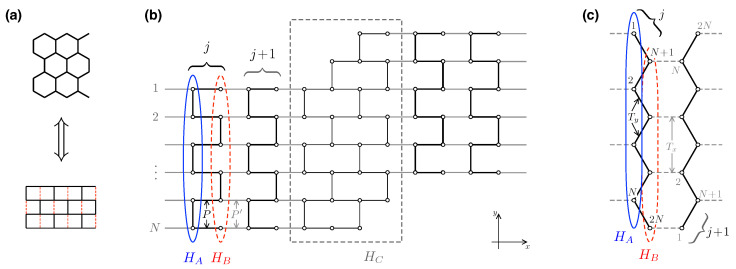
(**a**) A nanosystem of 24 sites carved out of the honeycomb lattice and an equivalent section of a square lattice with every second vertical bond removed (red dashed lines). (**b**) Schematic of an *open* system in computer simulation. Semi-infinite leads (with *zigzag* edges) are built from identical sections (the *j*-th and j+1 sections are marked for the left lead), each consisting of two subsections *A* and *B*, coupled only by horizontal bonds contributing to the matrix blocks *P* and $P^{\prime }$. Leads contact the central section (surrounded by dashed rectangle) of a generic shape. (**c**) Analogous decomposition and the numbering scheme for the lead with *armchair edges* (see main text for details).

## Data Availability

Data are contained within the article.

## Appendix A. Numerical Mode-Matching for the Honeycomb Lattice

### Appendix A.1. The Nanosystem

In the following section, we show how the computational method originally developed by Ando for a square lattice [96] can be adapted to the honeycomb lattice. The key point is to notice that—when discussed on the level of tight-binding Hamiltonian (63)—a honeycomb lattice with nearest-neighbor hoppings only becomes equivalent to a square lattice with some bonds removed (see Figure A1a). The reasoning presented here can, with simple modifications, be applied to a honeycomb, square, or even triangular lattice (the last case may be applicable when discussing the recently discovered two-dimensional form of gold, *goldenene*; see Ref. [106]).

The nanosystem, shown schematically in Figure A1b, represents a generic case that is tractable within the Landauer–Büttiker formalism. Two semi-infinite leads are built from repeatable sections, allowing one to find the normal modes (propagating and evanescent) that define the basis for the scattering matrix—with moderate computational effort—via the secular equation (see next Appendix A.2); the central part may have an arbitrary shape, and thus the mode matching must be performed numerically on the lattice, which becomes a case of solving a sparse linear system of equations.

The details (and remaining assumptions) of both computational steps are given below.

### Appendix A.2. Solutions in the Leads

Keeping in mind the analogy between the honeycomb lattice and the square lattice mentioned above, we now consider an infinitely long wire with a width of *N* sites, short sections of which are schematically shown in Figure A1b (for zigzag edges) and Figure A1c (for armchair edges). The quantum-mechanical equation of motion can be written as

(A1) $$T^{†}\varphi_{j-1}+H_{0}\varphi_{j}+T\varphi_{j+1}=E\varphi_{j},$$

where

(A2) $$\varphi_{j}=\left(\begin{array}{c} \varphi_{A,j}\\ \varphi_{B,j} \end{array}\right)$$

is the 2N-component wavefunction with the probability amplitudes corresponding to the sites in the *A* and *B* blocks; i.e., $\varphi_{A(B),j}={\left[\varphi_{A(B),j}^{\left(1\right)},\cdots ,\varphi_{A(B),j}^{\left(N\right)}\right]}^{T}$. It is worth noting that the lead width in physical units, $W_{\infty }$, is related to *N* as follows

(A3) $$W_{\infty }=Na\times \left\{\begin{array}{cc} \frac{1}{2}\sqrt{3} & (\mathrm{zigzag}\;\mathrm{edges}),\\ 1 & (\mathrm{armchair}\;\mathrm{edges}). \end{array}\right.$$

Therefore, $W_{\infty }$ can be interpreted as a circumference of a nanotube that can be created by connecting the edge sites at each section by additional (vertical) hopping.

For *zigzag edges*, $H_{0}$ and *T* are 2N×2N matrices with the following block structure

(A4) $$H_{0}^{\left(\mathrm{zig}\right)}=\left(\begin{array}{cc} H_{A}^{z} & P\\ P^{†} & H_{B}^{z} \end{array}\right),\;\;\;\;\;\;T^{\left(\mathrm{zig}\right)}=\left(\begin{array}{cc} 0 & 0\\ P^{\prime } & 0 \end{array}\right).$$

The block matrices $H_{A}^{z}\neq H_{B}^{z}$ contain values of the electostatic potential energy (i.e., $-V_{0}$ for all sites) and *vertical* hopping elements depicted in Figure A1; they are tridiagonal matrices with every second hopping removed, namely

(A5) $$\begin{array}{cc} {\left(H_{A}^{z}\right)}_{ll^{\prime }} & =-V_{0}\delta_{l,l^{\prime }}-t_{0}\left(\delta_{l,l^{\prime }-1}+\delta_{l-1,l^{\prime }}\right)\left[l\;\mathrm{mod}\;2\right], \end{array}$$

(A6) $$\begin{array}{cc} {\left(H_{B}^{z}\right)}_{ll^{\prime }} & =-V_{0}\delta_{l,l^{\prime }}-t_{0}\left(\delta_{l,l^{\prime }-1}+\delta_{l-1,l^{\prime }}\right)\left[\left(l+1\right)\;\mathrm{mod}\;2\right], \end{array}$$

where $l,l^{\prime }=1\ldots N$, and $\delta_{ll^{\prime }}$ is the Kronecker delta. The matrices *P* and $P^{\prime }$ contain horizontal hoppings; for the zero magnetic field, we have

(A7) $${\left(P\right)}_{ll^{\prime }}={\left(P^{\prime }\right)}_{ll^{\prime }}=-t_{0}\delta_{l,l^{\prime }}.$$

For a uniform magnetic field, the Peierls substitution can be applied; it reads as $P_{ll}\to P_{ll}\exp\left(2\pi il\Phi_{a}/\Phi_{0}\right)$ (l=1…N), with $\Phi_{a}$ the flux per unit cell and $\Phi_{0}=h/e$ the flux quantum (the generalization for some geometric strains, leading to position-dependent hopping is also possible, provided that the invariance upon j→j+1—see Equation (A1)—is preserved).

For *armchair edges*, the site-numbering scheme presented in Figure A1c allows us to keep the block structure of Equation (A1), with

(A8) $$H_{0}^{\left(\mathrm{arm}\right)}=\left(\begin{array}{cc} H_{A}^{a} & T_{y}\\ T_{y}^{†} & H_{B}^{a} \end{array}\right),\;\;\;\;\;\;T^{\left(\mathrm{arm}\right)}=\left(\begin{array}{cc} 0 & 0\\ T_{x} & 0 \end{array}\right).$$

Now, the matrices $H_{A}^{a}=H_{B}^{a}$ contain only diagonal elements

(A9) $${\left(H_{A}^{a}\right)}_{ll^{\prime }}={\left(H_{B}^{a}\right)}_{ll^{\prime }}=-V_{0}\delta_{l,l^{\prime }}.$$

The remaining blocks, $T_{x}$ and $T_{y}$, are given by

(A10) $$\begin{array}{cc} {\left(T_{x}\right)}_{ll^{\prime }} & =-t_{0}\delta_{l,N-l^{\prime }+1} \end{array}$$

(A11) $$\begin{array}{cc} {\left(T_{y}\right)}_{ll^{\prime }} & =-t_{0}\left(\delta_{l,l^{\prime }}+\delta_{l-1,l^{\prime }}\right), \end{array}$$

where $l,l^{\prime }=1\ldots N$ again.

Using the familiar Bloch ansatz

(A12) $$\varphi_{j}=\lambda^{j}\varphi_{0},$$

we arrive to the secular equation for zigzag edges

(A13) $${\left(\begin{array}{cc} H_{A}^{z}\;-\;EI & P\\ P^{\prime } & 0 \end{array}\right)}^{-1}\left(\begin{array}{cc} 0 & -{P^{\prime }}^{†}\\ -P^{†} & EI\;-\;H_{B}^{z} \end{array}\right)\varphi_{0}=\lambda \varphi_{0},$$

where I is the N×N identity matrix and the eigenvalues λ are complex with |λ|=1 (for *propagating modes*) or real with |λ|≠1 (for *evanescent* modes). For armchair edges, Equation (A13) holds with the following substitutions

(A14) $$H_{A}^{z}\to H_{A}^{a},\;\;\;H_{B}^{z}\to H_{B}^{a},\;\;\;P\to T_{y},\;\;\;P^{\prime }\to T_{x}.$$

If the generalization involving the dependence of the matrix elements on the position across the lead, such as the Peierls substitution mentioned above, is desired, one must double the number blocks in Equation (A8) by introducing $\varphi_{j}={\left(\varphi_{A1,j},\varphi_{B1,j},\varphi_{A2,j},\varphi_{B2,j}\right)}^{T}$; however, such a case is beyond the scope of the present paper.

### Appendix A.3. Basis for the Scattering Matrix

The secular equation derived above, given explicitly by Equation (A13) for zigzag edges (z.e.), with the necessary substitutions for armchair edges (a.e.) in Equation (A14), can be diagonalized numerically using standard software packages in order to find the full set of eigenvalues (λ) with corresponding right-eigenvectors $\varphi_{0}^{\left(\lambda \right)}$. We chose the double precision LAPACK routines dgemm and dgeev; see Ref. [107].

To correctly define the scattering matrix, the propagating modes must be normalized so that they carry equal currents along the main axis of each lead (i.e., the *x*-axis direction in Figure A1).

In general, the total current incoming at the *i*-th lattice site of the system described by the tight-binding Hamiltonian—see Equations (63) and (64) in the main text—is given by the time derivative of the charge

(A15) $${\dot{n}}_{i}=i\left[H,n_{i}\right]=i\sum_{j(s)}\left(t_{ij}c_{i}^{†}c_{j}-t_{ij}^{★}c_{j}^{†}c_{i}\right),$$

where j(i) runs over the nearest neighbors of *i*, complex hopping (i.e., $t_{ij}\neq t_{ij}^{★}$) is allowed, and the spin is omitted for clarity. In turn, the current flowing from site *i* to site *j* is described by the quantum-mechanical operator

(A16) $$J_{ij}=i\left(t_{ij}c_{i}^{†}c_{j}-t_{ij}^{★}c_{j}^{†}c_{i}\right).$$

Taking into account all the currents incoming and outgoing from a repeatable section (*j*) of the lead (see Figure A1) via individual bonds, projected onto the *x*-direction, brings us to the total *x*-current operator $J_{x}$, which—when acting on the right-eigenvector $\varphi_{0}^{\left(\lambda \right)}$ (see Equations (A13) and (A14)) corresponding to the eigenvalue λ—is equivalent to

(A17) $$J_{x}^{\left(\lambda \right)}=i\left(\begin{array}{cc} 0 & -\lambda^{-1}{P^{\prime }}^{†}\\ \lambda P^{\prime } & 0 \end{array}\right)\;\;\;\;\left(z.e.\right),$$

or

(A18) $$J_{x}^{\left(\lambda \right)}=i\left(\begin{array}{cc} 0 & -\lambda^{-1}T_{x}^{†}\\ \lambda T_{x} & 0 \end{array}\right)\;\;\;\;\left(a.e.\right).$$

Subsequently, the normalized eigenvector can be written as

(A19) $$v_{\lambda }^{\mathrm{pro}}=\frac{1}{\sqrt{\left|{\varphi_{0}^{\left(\lambda \right)}}^{†}J_{x}^{\left(\lambda \right)}\varphi_{0}^{\left(\lambda \right)}\right|}}\;\varphi_{0}^{\left(\lambda \right)}\;\;\;\;\left(\mathrm{for}\;\;|\lambda |=1\right),$$

and the sign

(A20) $$s_{\lambda }\equiv {\left(v_{\lambda }^{\mathrm{pro}}\right)}^{†}J_{x}^{\left(\lambda \right)}v_{\lambda }^{\mathrm{pro}}=\pm 1$$

identifies the direction of propagation. For evanescent modes, the normalization is irrelevant, and we simply set

(A21) $$v_{\lambda }^{\mathrm{eva}}=\varphi_{0}^{\left(\lambda \right)}\;\;\;\;\left(\mathrm{for}\;\;|\lambda |\neq 1\right).$$

In analogy to propagating modes, |λ|>1 now identifies the evanescent mode decaying to the left, while |λ|<1 identifies the evanescent mode decaying to the right. The real-space components of the normalized eigenvector for the *j*-th section of the lead can be written as column vectors of the dimension 2N, namely

(A22) $$v_{\lambda ,j}^{\mathrm{pro}}=\lambda^{j}v_{\lambda }^{\mathrm{pro}},\;\;\;\;\;\;v_{\lambda ,j}^{\mathrm{eva}}=\lambda^{j}v_{\lambda }^{\mathrm{eva}}.$$

Now it is worth generalizing the discussion slightly by allowing the left and right leads to not be necessarily identical, i.e., they may have different numbers of sites across $N^{\left(L\right)}$ and $N^{\left(R\right)}$, or different electostatic potential energy levels $-V_{0}^{L}$ and $-V_{0}^{R}$. In such a case, the secular equation, Equation (A13), appears in the two versions, with the block matrices replaced by

(A23) $$\begin{array}{cc} H_{A} & \to H_{A}^{\alpha },\;\;H_{B}\to H_{B}^{\alpha },\;\;P\to P_{\alpha },\;\;P^{\prime }\to P_{\alpha }^{\prime },\\ \; & \mathrm{for}\;\;\alpha =L,R, \end{array}$$

leading to (a priori) different numbers of propagating modes per single direction, $N_{\mathrm{pro}}^{\left(L\right)}$ and $N_{\mathrm{pro}}^{\left(R\right)}$. The number of left- (or right-) decaying evanescent modes is then given by

(A24) $$N_{\mathrm{eva}}^{\left(\alpha \right)}=N^{\left(\alpha \right)}-N_{\mathrm{pro}}^{\left(\alpha \right)},\;\;\;\;\mathrm{for}\;\;\alpha =L,R.$$

Therefore, the number of elements (i.e., normalized eigenvectors) for the following four sets of (left-, right-) propagating and (left-, right-) decaying modes in a single electrode are as follows

(A25) $$\begin{array}{cc} \#\left\{v_{\lambda ,j}^{\alpha ,\mathrm{pro}},s_{\lambda }>0\right\} & =\#\left\{v_{\lambda ,j}^{\alpha ,\mathrm{pro}},s_{\lambda }<0\right\}=N_{\mathrm{pro}}^{\left(\alpha \right)}, \end{array}$$

(A26) $$\begin{array}{cc} \#\left\{v_{\lambda ,j}^{\alpha ,\mathrm{eva}},\left|\lambda \right|>1\right\} & =\#\left\{v_{\lambda ,j}^{\alpha ,\mathrm{eva}},\left|\lambda \right|<1\right\}=N^{\left(\alpha \right)}\;-\;N_{\mathrm{pro}}^{\left(\alpha \right)},\\ \; & \mathrm{for}\;\;\alpha =L,R. \end{array}$$

For further considerations, we define the matrices $W_{j}^{\left(L\right)}$ and $W_{j}^{\left(R\right)}$, in which the selected eigenvectors are stored column by column, i.e.,

(A27) $$\begin{array}{c} W_{j}^{\left(L\right)}=\left(\begin{array}{c} W_{A,j}^{\left(L\right)}\\ W_{B,j}^{\left(L\right)} \end{array}\right)=\left(\left\{v_{\lambda ,j}^{L,\mathrm{pro}},s_{\lambda }>0\right\};\left\{v_{\lambda ,j}^{L,\mathrm{pro}},s_{\lambda }<0\right\};\left\{v_{\lambda ,j}^{L,\mathrm{eva}},\left|\lambda \right|>1\right\}\right)\\ \equiv \left(\begin{array}{cc} V_{A,j}^{\left(L\right)} & U_{A,j}^{\left(L\right)}\\ V_{B,j}^{\left(L\right)} & U_{A,j}^{\left(L\right)} \end{array}\right), \end{array}$$

and

(A28) $$\begin{array}{c} W_{j}^{\left(R\right)}=\left(\begin{array}{c} W_{A,j}^{\left(R\right)}\\ W_{B,j}^{\left(R\right)} \end{array}\right)=\left(\left\{v_{\lambda ,j}^{R,\mathrm{eva}},\left|\lambda \right|<1\right\};\left\{v_{\lambda ,j}^{R,\mathrm{pro}},s_{\lambda }>0\right\};\left\{v_{\lambda ,j}^{R,\mathrm{pro}},s_{\lambda }<0\right\}\right)\\ \equiv \left(\begin{array}{cc} U_{A,j}^{\left(R\right)} & V_{A,j}^{\left(R\right)}\\ U_{B,j}^{\left(R\right)} & V_{A,j}^{\left(R\right)} \end{array}\right). \end{array}$$

In the above, the blocks $W_{A,j}^{\left(\alpha \right)}$ and $W_{B,j}^{\left(\alpha \right)}$ store the matrix rows $1\ldots N^{\left(\alpha \right)}$ and $N^{\left(\alpha \right)}+1\ldots 2N^{\left(\alpha \right)}$, respectively, for α=L,R. These blocks correspond to the first $N^{\left(\alpha \right)}$ sites ($W_{A,j}^{\left(\alpha \right)}$) and to the next $N^{\left(\alpha \right)}$ sites ($W_{B,j}^{\left(\alpha \right)}$) in the *j*-th section of a single lead.

The real-space components of an arbitrary wavefunction in the *j*-th section of the (left,right) lead can now be represented as

(A29) $$\begin{array}{c} \Psi_{j}^{\left(L\right)}=W_{j}^{\left(L\right)}{\left(\left\{a_{\lambda ,+}^{L,\mathrm{pro}}\right\};\left\{b_{\lambda ,-}^{L,\mathrm{pro}}\right\};\left\{b_{\lambda ,-}^{L,\mathrm{eva}}\right\}\right)}^{T}, \end{array}$$

(A30) $$\begin{array}{c} \Psi_{j}^{\left(R\right)}=W_{j}^{\left(R\right)}{\left(\left\{b_{\lambda ,+}^{R,\mathrm{eva}}\right\};\left\{b_{\lambda ,+}^{R,\mathrm{pro}}\right\};\left\{a_{\lambda ,-}^{R,\mathrm{pro}}\right\}\right)}^{T}, \end{array}$$

where each set of complex coefficients, $\left\{a_{\lambda ,+}^{L,\mathrm{pro}}\right\}$, etc., corresponds to the matching set of eigenvectors in the matrix $W_{j}^{\left(L\right)}$ or $W_{j}^{\left(R\right)}$. Parts of the matrices $W_{j}^{\left(\alpha \right)}$, α=L,R, containing the columns corresponding to the coefficients $b_{\lambda ,\mp }^{\alpha ,\mathrm{pro}}$, $b_{\lambda ,\mp }^{\alpha ,\mathrm{eva}}$, read

(A31) $$\begin{array}{cc} \left(\begin{array}{c} U_{A,j}^{\left(L\right)}\\ U_{B,j}^{\left(L\right)} \end{array}\right)\; & =\left(\left\{v_{\lambda ,j}^{L,\mathrm{pro}},s_{\lambda }<0\right\};\left\{v_{\lambda ,j}^{L,\mathrm{eva}},\left|\lambda \right|>1\right\}\right), \end{array}$$

(A32) $$\begin{array}{cc} \left(\begin{array}{c} U_{A,j}^{\left(R\right)}\\ U_{B,j}^{\left(R\right)} \end{array}\right)\; & =\left(\left\{v_{\lambda ,j}^{R,\mathrm{eva}},\left|\lambda \right|<1\right\};\left\{v_{\lambda ,j}^{R,\mathrm{pro}},s_{\lambda }>0\right\}\right), \end{array}$$

where each of the blocks $U_{A,j}^{\left(\alpha \right)}$, $U_{B,j}^{\left(\alpha \right)}$ is a square matrix of dimension $N^{\left(\alpha \right)}\times N^{\left(\alpha \right)}$. The remaining columns, corresponding to the coefficients $a_{\lambda ,\pm }^{\alpha ,\mathrm{pro}}$, define the matrices

(A33) $$\begin{array}{cc} \left(\begin{array}{c} V_{A,j}^{\left(L\right)}\\ V_{B,j}^{\left(L\right)} \end{array}\right)\; & =\left(\left\{v_{\lambda ,j}^{L,\mathrm{pro}},s_{\lambda }>0\right\}\right), \end{array}$$

(A34) $$\begin{array}{cc} \left(\begin{array}{c} V_{A,j}^{\left(R\right)}\\ V_{B,j}^{\left(R\right)} \end{array}\right)\; & =\left(\left\{v_{\lambda ,j}^{R,\mathrm{pro}},s_{\lambda }<0\right\}\right), \end{array}$$

where the dimensions of the blocks $V_{A,j}^{\left(\alpha \right)}$, $V_{B,j}^{\left(\alpha \right)}$ are $N^{\left(\alpha \right)}\times N_{\mathrm{pro}}^{\left(\alpha \right)}$, for α=L,R.

### Appendix A.4. The Scattering Problem

In the last part of this Appendix, we will use the notation relevant for the leads with zigzag edges (see Figure A1). Nevertheless, if a version of the approach for the leads with armchair edges is desired, it can be easily generated by performing the substitutions given by Equation (A14) (we emphasize here that the central section of the system, described by the tight-binding Hamiltonian $H_{C}$, may have an arbitrary shape so that the irregular edges in the central section are also tractable within the presented approach.)

Using the definitions introduced in the previous subsections, we can now write down the quantum-mechanical equation of motion for the entire nanosystem as follows

(A35) $$\begin{array}{c} \left(\begin{array}{ccccc} {P_{L}}^{†}W_{A,-1}^{\left(L\right)}+\left(H_{B}^{L}\;-\;E\right)W_{B,-1}^{\left(L\right)} & \;\;\vec{P_{L}^{\prime }}\;\; & & & \\ {\vec{P_{L}^{\prime }}}^{†}W_{B,-1}^{\left(L\right)} & & & & \\ & & H_{C}-E & & \\ & & & & \overset{\leftarrow }{P_{R}^{\prime }}W_{A,1}^{\left(R\right)}\\ & & & \;\;{\overset{\leftarrow }{P_{R}^{\prime }}}^{†}\;\; & \left(H_{A}^{R}\;-\;E\right)W_{A,1}^{\left(R\right)}+P_{R}W_{B,1}^{\left(R\right)} \end{array}\right)\\ \times \left(\begin{array}{c} \left\{a_{\lambda ,+}^{L,\mathrm{pro}}\right\}\\ \left\{b_{\lambda ,-}^{L,\mathrm{pro}}\right\}\\ \left\{b_{\lambda ,-}^{L,\mathrm{eva}}\right\}\\ \Psi_{C}\\ \left\{b_{\lambda ,+}^{R,\mathrm{eva}}\right\}\\ \left\{b_{\lambda ,+}^{R,\mathrm{pro}}\right\}\\ \left\{a_{\lambda ,-}^{R,\mathrm{pro}}\right\} \end{array}\right)=0, \end{array}$$

where we set j=−1 for the terminal section of the left lead and j=1 for the terminal section of the right lead. The unit matrix is omitted in expressions $\left(H_{B}^{L}-EI\right)$ and $\left(H_{A}^{R}-EI\right)$; the coefficients in each set, $\left\{a_{\lambda ,+}^{L,\mathrm{pro}}\right\}$, etc., are now listed as columns; $\Psi_{C}$ is a column vector that stores the wavefunction amplitudes for the central section.

In a case when the width of the central section, $N_{C}$, exceeds the width of (at least) one of the leads, i.e., $N_{C}>N^{\left(L\right)}$ or $N_{C}>N^{\left(R\right)}$ (in addition, the leads may be vertically displaced, as in the nanosystem shown in Figure A1), the blocks occurring near the upper-left or lower-right corner of the main matrix in Equation (A35) that connect the central section with the leads, namely, $\vec{P_{L}^{\prime }}$ and $\overset{\leftarrow }{P_{R}^{\prime }}$ with their hermitian conjugates ${\vec{P_{L}^{\prime }}}^{†}$, ${\overset{\leftarrow }{P_{R}^{\prime }}}^{†}$, are constructed in such a way that the original matrix $P_{\alpha }^{\prime }$ is horizontally or vertically expanded and filled with zeros, namely

(A36) $$\vec{P_{L}^{\prime }}=\left(\begin{array}{ccc} 0 & P_{L}^{\prime } & 0 \end{array}\right),\;\;\;\;\;\;\overset{\leftarrow }{P_{R}^{\prime }}=\left(\begin{array}{c} 0\\ P_{R}^{\prime }\\ 0 \end{array}\right),$$

where the elements in the original block match the existing bonds connecting the lattice sites (for $N_{C}=N^{\left(L\right)}=N^{\left(R\right)}$, we simply have $\vec{P_{L}^{\prime }}=P_{L}^{\prime }$ and $\overset{\leftarrow }{P_{R}^{\prime }}=P_{R}^{\prime }$.)

In addition, Equation (A13) with substitutions given by Equation (A23) for the two leads, guarantees that

(A37) $$\begin{array}{cc} {P_{L}}^{†}W_{A,-1}^{\left(L\right)}+\left(H_{B}^{L}\;-\;E\right)W_{B,-1}^{\left(L\right)} & =-P_{L}^{\prime }W_{A,0}^{\left(L\right)}, \end{array}$$

(A38) $$\begin{array}{cc} \left(H_{A}^{R}\;-\;E\right)W_{A,1}^{\left(R\right)}+P_{R}W_{B,1}^{\left(R\right)} & =-{P_{R}^{\prime }}^{†}W_{B,0}^{\left(R\right)}, \end{array}$$

allowing some further simplification of Equation (A35).

We now introduce the $\left(N_{\mathrm{pro}}^{\left(L\right)}+N_{\mathrm{pro}}^{\left(R\right)}\right)\times \left(N_{\mathrm{pro}}^{\left(L\right)}+N_{\mathrm{pro}}^{\left(R\right)}\right)$ scattering matrix

(A39) $$S=\left(\begin{array}{cc} r & t^{\prime }\\ t & r^{\prime } \end{array}\right),$$

defined in such a way that

(A40) $$\left(\begin{array}{c} \left\{b_{\lambda ,-}^{L,\mathrm{pro}}\right\}\\ \left\{b_{\lambda ,+}^{R,\mathrm{pro}}\right\} \end{array}\right)=S\left(\begin{array}{c} \left\{a_{\lambda ,+}^{L,\mathrm{pro}}\right\}\\ \left\{a_{\lambda ,-}^{R,\mathrm{pro}}\right\} \end{array}\right).$$

If we assume that the coefficients $\left\{a_{\lambda ,+}^{L,\mathrm{pro}}\right\}$ and $\left\{a_{\lambda ,-}^{R,\mathrm{pro}}\right\}$ are independent amplitudes of the incoming waves in the left and right leads, respectively, we can transform the linear system in Equation (A35) into a series of Kramers systems, one for each incoming mode *l*; namely,

(A41) −PL′UA,0(L)PL′→PL′→†UB,−1(L)HC−EPR′←UA,1(R)PR′←†−PR′†UB,0(R)rt′      ΨC(l)tr′=PL′VA,0(L)0−PL′→†VB,−1(L)0                   00−PR′←VA,1(R)0PR′†VB,0(R),

where $\left\{\Psi_{C}^{\left(l\right)}\right\}$ is the set of wavefunctions for the central section, for $l=1\ldots N_{\mathrm{pro}}^{\left(L\right)}+N_{\mathrm{pro}}^{\left(R\right)}$, stored as columns. In the above, we used Equations (A37) and (A38).

The rank of the main matrix in Equation (A41) is equal to $N_{\mathrm{pro}}^{\left(L\right)}+2N_{C}M+N_{\mathrm{pro}}^{\left(R\right)}$, where *M* denotes the number of vertical sections, each containing (up to) $2N_{C}$ lattice sites. Although the shape of the central section is—in principle—irregular, a site-numbering scheme analogous to that used for the leads can be applied. In the case of a considerable number of disconnected lattice sites, an additional enumeration for the central part can be applied to eliminate the matrix elements corresponding to disconnected sites and to reduce the rank of the main matrix.

For the total number of sites $2N_{C}M<5\times 10^{5}$, such as in the numerical examples presented in the main text, one can use standard linear algebra software to solve the linear system given by Equation (A41) numerically and find the unknowns, including all elements of the scattering matrix *S* (A39). We used the double precision LAPACK routine zgbsv—see Ref. [107]—which employs the band storage scheme for the main matrix with $2N_{C}-1$ subdiagonals and $2N_{C}-1$ superdiagonals. Assuming that the proportions of the middle section are not very different from those of the square, i.e., $N_{C}∼M∼\sqrt{N_{\mathrm{tot}}}$ (with the total number of sites $N_{\mathrm{tot}}$) we can estimate the computational complexity to be $O(N_{\mathrm{tot}}^{2})$, which is equivalent to the complexity of the more commonly used recursive Green’s function method [108].

Since the normal modes in the left and right leads are calculated independently, we can validate the final numerical output by checking the unitarity condition for the scattering matrix, $SS^{†}=S^{†}S=I$. The deviation from unitarity is quantified by

(A42) $$\varepsilon_{S}=\max\left\{\left|{\left(S^{†}S\right)}_{ll^{\prime }}-\delta_{ll^{\prime }}\right|,\;1⩽l,l^{\prime }⩽N_{\mathrm{pro}}^{\left(L\right)}\;+\;N_{\mathrm{pro}}^{\left(R\right)}\right\}.$$

In the numerical problems considered here, the above does not exceed $\varepsilon_{S}≲10^{-6}$; the maximal value is approached for the half-disk system, for which the number of propagating modes in the right (i.e., the wider) lead is $52⩽N_{\mathrm{pro}}^{\left(R\right)}⩽537$ for the energy range of $0.1⩽\left(E+V_{0}\right)/t_{0}⩽0.9$.

If more than two leads contact the central system, a generalization is straightforward, provided that we have a group of (one or more) parallel left leads and a group of parallel right leads. In such a case, the block structure of Equation (A41) is preserved, and it is only necessary to modify the contents of the blocks connecting the central section and the leads accordingly. For some more complex cases, relevant graph algorithms are implemented in the KWANT package [109].

For much larger systems, the direct lattice approach presented above must be replaced by the method truncating the wavefunction within orthogonal polynomials, such as that recently implemented in the KITE software [110].
